# The genetic basis of 3-hydroxypropanoate metabolism in *Cupriavidus necator* H16

**DOI:** 10.1186/s13068-019-1489-5

**Published:** 2019-06-17

**Authors:** Christian Arenas-López, Jessica Locker, Diego Orol, Frederik Walter, Tobias Busche, Jörn Kalinowski, Nigel P. Minton, Katalin Kovács, Klaus Winzer

**Affiliations:** 1 BBSRC/EPSCR Synthetic Biology Research Centre (SBRC), School of Life Sciences, Centre for Biomolecular Sciences, University of Nottingham, Nottingham, NG7 2RD UK; 2 Center for Biotechnology (CeBiTec), Bielefeld University, Universitätsstraße 27, 33615 Bielefeld, Germany

**Keywords:** 3-Hydroxypropionic acid, Metabolic engineering, *Cupriavidus necator*, *Ralstonia eutropha*, Co-metabolism, Carbon fixation, Malonate semialdehyde dehydrogenase, β-alanine, Valine

## Abstract

**Background:**

3-Hydroxypropionic acid (3-HP) is a promising platform chemical with various industrial applications. Several metabolic routes to produce 3-HP from organic substrates such as sugars or glycerol have been implemented in yeast, enterobacterial species and other microorganisms. In this study, the native 3-HP metabolism of *Cupriavidus necator* was investigated and manipulated as it represents a promising chassis for the production of 3-HP and other fatty acid derivatives from CO_2_ and H_2_.

**Results:**

When testing *C. necator* for its tolerance towards 3-HP, it was noted that it could utilise the compound as the sole source of carbon and energy, a highly undesirable trait in the context of biological 3-HP production which required elimination. Inactivation of the methylcitrate pathway needed for propionate utilisation did not affect the organism’s ability to grow on 3-HP. Putative genes involved in 3-HP degradation were identified by bioinformatics means and confirmed by transcriptomic analyses, the latter revealing considerably increased expression in the presence of 3-HP. Genes identified in this manner encoded three putative (methyl)malonate semialdehyde dehydrogenases (*mmsA1, mmsA2* and *mmsA3*) and two putative dehydrogenases (*hpdH* and *hbdH*). These genes, which are part of three separate *mmsA* operons, were inactivated through deletion of the entire coding region, either singly or in various combinations, to engineer strains unable to grow on 3-HP. Whilst inactivation of single genes or double deletions could only delay but not abolish growth, a triple ∆*mmsA1*∆*mmsA2*∆*mmsA3* knock-out strain was unable utilise 3-HP as the sole source of carbon and energy. Under the used conditions this strain was also unable to co–metabolise 3-HP alongside other carbon and energy sources such as fructose and CO_2_/H_2_. Further analysis suggested primary roles for the different *mmsA* operons in the utilisation of β-alanine generating substrates (*mmsA1)*, degradation of 3-HP (*mmsA2)*, and breakdown of valine (*mmsA3)*.

**Conclusions:**

Three different (methyl)malonate semialdehyde dehydrogenases contribute to 3-HP breakdown in *C. necator* H16. The created triple ∆*mmsA1*∆*mmsA2*∆*mmsA3* knock-out strain represents an ideal chassis for autotrophic 3-HP production.

**Electronic supplementary material:**

The online version of this article (10.1186/s13068-019-1489-5) contains supplementary material, which is available to authorized users.

## Introduction

*Cupriavidus necator* (formerly *Ralstonia eutropha*) strain H16 is a well-studied β-proteobacterium capable of autotrophic, heterotrophic and mixotrophic growth [1]. Due to its ability to grow chemolithoautotrophically on CO_2_ and H_2_, and to store vast amounts of fixed carbon in the form of polyhydroxybutyrate (PHB), it is a promising candidate for the sustainable production of chemical commodities from waste gases [2–4]. When growing heterotrophically, it can utilise a wide range of organic compounds as the sole sources of carbon and energy [5, 6]. Although catabolism of carbohydrate derivatives is limited to the utilisation of fructose, gluconate and *N*-acetylglucosamine, the organism is able to grow on fatty acids, alcohols, tricarboxylic acid cycle intermediates, oleic acid derivatives, and aromatic compounds [7–10]. Short chain fatty acids such as acetic acid, butyric acid and propionic acid, as well as hydroxylated fatty acids such as 3-hydroxypropionic acid can either support growth or be incorporated into polyhydroxyalkanoates (PHAs) under certain conditions [11–13].

3-Hydroxypropionic acid (3-HP) is a promising platform chemical with multiple industrial applications, including the conversion to acrylic acid, acrylamide, propandiol and 3-HP polymers. It is one of the top 12 value-added chemicals which can be derived from biomass, according to a study by the United States Department of Energy [14]. When considering biological production of chemical commodities such as 3-HP, it is important to ensure that the generated compounds are not re-metabolised by the producing organism at any stage during the process. This might be achieved by either metabolic or process engineering. In this work, we investigated the native 3-HP metabolism in *C. necator* strain H16 with the aim of engineering a strain incapable of 3-HP utilisation, to be used as a chassis for the future introduction of biosynthetic routes towards its production.

There are two main pathways that have been proposed for assimilation of 3-HP in different organisms: a reductive route and an oxidative route [15–18]. The former is CoA-dependent and comprises the reductive conversion of 3-HP to propionyl-CoA via the intermediates 3-hydroxypropionyl-CoA and acrylyl-CoA. This pathway has been well studied in the phototrophic organism *Chloroflexus aurantiacus* where a propionyl-Coenzyme A synthase carries out the above reaction sequence as part of the 3-hydroxypropionate cycle for CO_2_ fixation [19]. Moreover, a similar route has been proposed for 3-HP assimilation in the photoheterotrophic bacterium *Rhodobacter sphaeroides* [15]. In this case, a candidate enzyme that most likely catalyses the reductive conversion of acrylyl-CoA to propionyl-CoA, Acul, has been identified [20]. In *C. necator* H16, a propionate CoA-transferase (Pct) has been identified and characterised. It exhibits similarities in terms of sequence and structure with Pct from *C. propionicum* and its broad substrate specificity resulted in its classification as a family I Coenzyme A transferase [21]. Using acetyl-CoA as a CoA donor, the list of carboxylic acids that can act as CoA acceptors in a Pct-catalysed reaction includes compounds such as propionate, butyrate, 3-HP, 3-hydroxybutyrate, crotonate, acrylate, and others [22]. Propionate was identified as the best substrate followed by 3-HP, 3-HB and acrylate [21]. More recently, in a study for the production of acrylic acid via 3-HP, 3-HP-CoA and acrylyl-CoA, Pct from *C. necator* H16 was identified as the most active CoA-transferase to catalyse the conversion from 3-HP to 3-HP-CoA among 14 candidates from other organisms [23]. This opened the possibility that 3-HP breakdown in *C. necator* could follow the reductive route via propionyl-CoA as proposed by Peplinski et al. [24], a hypothesis further supported by the observation that propionate is readily degraded by the organism via the methylcitric acid pathway [25].

On the other hand, in the much shorter CoA-independent route, 3-HP would be oxidised to malonate semialdehyde, which would then be decarboxylated and thus converted to acetyl-CoA, ready to enter intermediary metabolism. Recent attempts to use engineered *Pseudomonas denitrificans* for the production of 3-HP from glycerol have shown that subsequent breakdown of the generated compound resulted in low yields [17, 26]. Specific inhibition of acid dehydrogenases completely blocked 3-HP assimilation, and detection of malonic and methyl malonic acids in samples analysed by GC/MS strengthened the hypothesis that breakdown might occur via the oxidative route [26]. Indeed, follow-up studies identified and characterised several dehydrogenases involved in the oxidation of 3-HP to malonate semialdehyde and their inactivation led to a strain unable to use 3-HP as sole carbon source [17, 27, 28]. Some of these genes have been shown to be organised in operons which are induced by 3-HP, including a (methyl)malonate semialdehyde dehydrogenase gene (*mmsA*) and a 3-hydroxyiso-butyrate dehydrogenase gene (*hbdH*) [29]. Findings made for other organisms such as an active 3-hydroxypropionate dehydrogenase in *Bacillus cereus* and a propionate degradation pathway (via 3-HP and malonate semialdehyde to acetyl-CoA) in *Candida albicans* suggest that the oxidative route is more widely used [16, 30].

Here, we report on the identification of several genes involved in 3-HP utilisation in *C. necator* H16, including three putative (methyl)malonate semialdehyde dehydrogenases and two putative dehydrogenases. The combined deletion of the former three genes resulted in a strain unable to grow on 3-HP as a source of carbon and energy.

## Results

### Utilisation of 3-HP as the sole carbon and energy source does not require the methylcitrate pathway

**Fig. 1:**
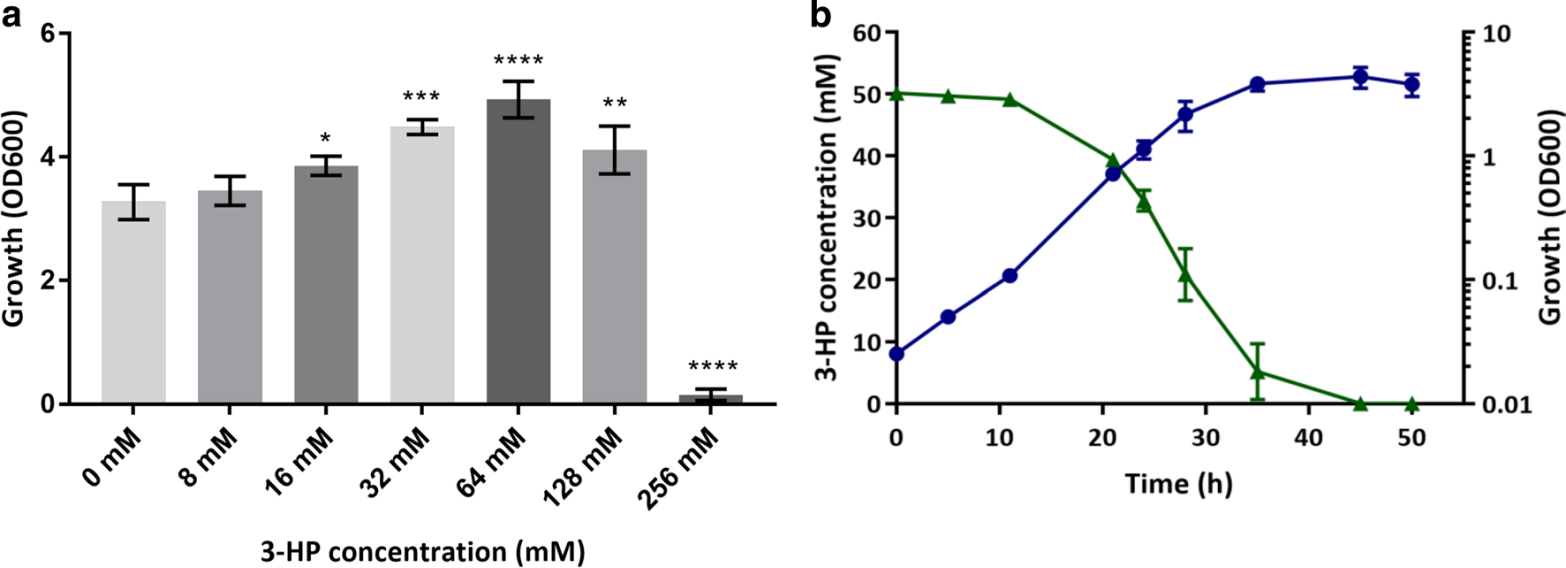
Growth of *C. necator* H16 on 3-HP together with fructose (**a**) and on 3-HP alone (**b**). **a** 24 h end point growth of *C. necator* H16 following cultivation in F-MM containing 0.4% (22 mM) fructose, and supplemented with increasing concentrations of 3-HP: 0 mM, 8 mM, 16 mM, 32 mM, 64 mM, 128 mM and 256 mM. Growth was determined in a BioLector microbioreactor system by correlating the measured scattered light values to an OD_600_ calibration curve. Error bars represent the standard deviation of the mean for three independent experiments. **** (*p* < 0.0001), *** (*p* = 0.0002), ** (*p* = 0.0041), * (*p* = 0.0479). **b** Growth of *C. necator* H16 with 50 mM 3-HP as the sole source of carbon and energy. Growth was followed by measuring OD_600_ (blue circles), 3-HP concentrations were quantified through HPLC (green triangles). Error bars represent the standard deviation of the mean for three independent experiments

When *C. necator* H16 was grown in minimal medium containing 0.4% fructose (F-MM) and supplemented with increasing concentrations of 3-HP (0–256 mM), higher final optical densities (ODs) were observed in the presence of 3-HP compared to cultures grown in F-MM alone (Fig. 1a). This suggested that 3-HP could be used as a carbon source in addition to fructose, unless provided at very high and presumably toxic concentrations (> 128 mM). To investigate this further, *C. necator* H16 was grown in minimal medium (MM) containing 50 mM 3-HP as the sole source of carbon and energy. Under these conditions, the provided 3-HP was completely consumed (Fig. 1b) confirming that it could support growth of the organism in MM.

It has been reported that *C. necator* H16 can activate 3-HP to 3-HP-CoA [21]. Furthermore, transcriptomic data from *C. necator* H16 cultivated on gluconate in presence of 3,3′-thiodipropionic acid (TDP) suggested that the generated TDP cleavage product 3-HP might be converted to propionyl-CoA and further metabolised to acetyl-CoA via the 2-methylcitrate pathway [24]. This hypothesis was tested by deleting the entire encoding gene cluster consisting of *prpR, prpB, prpC, acnM, orf5* and *prpD,* previously shown to be required for propionate metabolism [25]. However, this deletion had very little effect on growth and consumption of 3-HP when provided as the sole source of carbon and energy (Additional file 1: Figure S1), whereas it completely abolished growth on propionate, in agreement with previous findings [25].

### Identification of *C. necator* H16 genes predicted to play a role in 3-HP metabolism

**Fig. 2:**
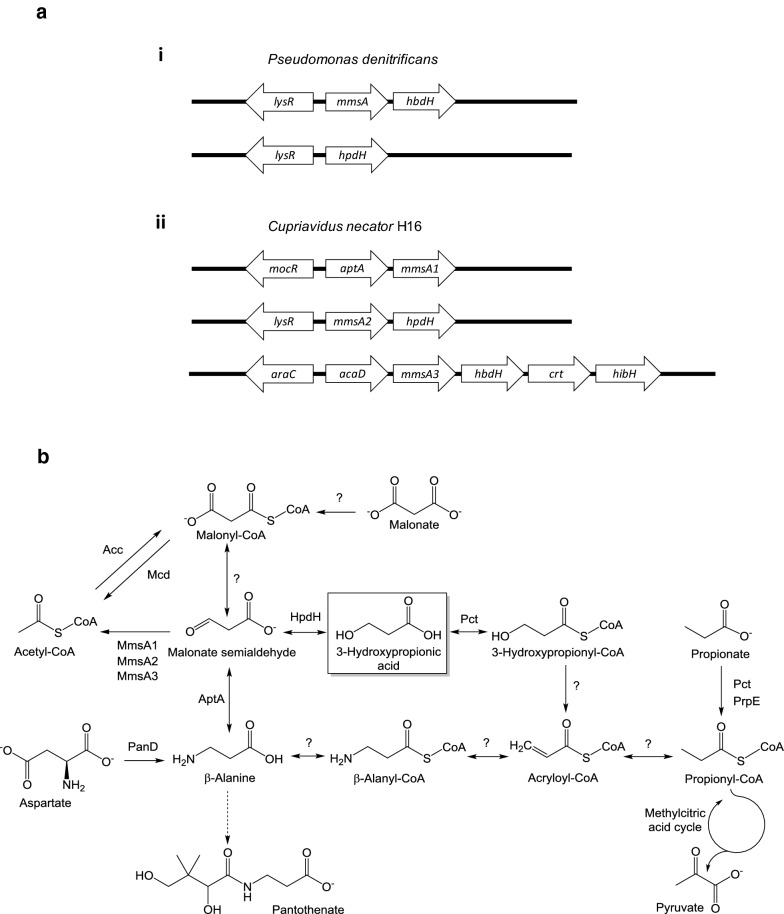
*Cupriavidus necator* genes involved on 3-HP degradation (**a**) and the C_3_ metabolic network (**b**). **a** Putative operons involved in 3-HP metabolism in *P. denitrificans* (i) and *C. necator* H16 (ii), together with the proposed upstream-located regulatory genes. Genes encode the following enzymes: (methyl)malonate semialdehyde dehydrogenase (*mmsA1*, H16_A0273; *mmsA2*, H16_A3664; *mmsA3*, H16_B1191), 3-hydroxy-propionate/iso-butyrate dehydrogenase (*hpdH*, H16_A3663; *hbdH*, H16_B1190), β-alanine pyruvate transaminase (*aptA*, H16_A0272), branched-chain acyl-CoA dehydrogenase (*acaD*, H16_B1192), enoyl-CoA dehydratase (*crt*, H16_B1189) and 3-hydroxyisobutyryl-CoA hydrolase (*hibH*; note: the gene is currently annotated to encode an enoyl-CoA hydratase/isomerase, H16_B1188). Divergently transcribed transcriptional regulator genes encode putative homologues of MocR (H16_A0271), LysR (H16_A3665) and AraC (H16_B1193), respectively. Note that only some of the encoded activities contribute to 3-HP metabolism as shown in (b). Sizes of genes and intergenic regions not drawn to scale. **b** Metabolism of 3-HP and related C_3_ compounds in *C. necator*. Acc, acetyl-CoA carboxylase; Mcd, malonate decarboxylase; PanD, l-aspartate decarboxylase; Pct, propionyl-CoA transferase; PrpE, propionyl-CoA synthase. For all other protein names see (**a**). Question marks indicate reactions for which *C. necator* enzymes may exist but have not been experimentally demonstrated

Given the continued consumption of 3-HP in the absence of a functional methylcitrate pathway, the H16 genome was interrogated for the presence of alternative 3-HP utilisation genes, including those encoding the CoA-independent route via malonate semialdehyde (Fig. 2). Indeed, three putative (methyl)malonate semialdehyde dehydrogenase genes, *mmsA1* (H16_A0273), *mmsA2* (H16_A3664) and *mmsA3* (H16_B1191), required for the oxidative decarboxylation of malonate semialdehyde to acetyl-CoA or, potentially, methylmalonate to propionyl-CoA, were found to be present in the genome and are annotated as such.

In *P. denitrificans*, 3-HP degradation genes are clustered in two separate operons each preceded by a divergently transcribed LysR-type regulator gene [17, 29]. The so-called C4 operon contains a (methyl)malonate semialdehyde dehydrogenase (*mmsA*) and a 3-hydroxyisobutyrate dehydrogenase gene (*hbdH*), whereas the C3 operon is monocistronic and consists of a single 3-hydroxypropionate dehydrogenase gene (*hpdH*). Screening of 3-HP-inducible systems in other microorganisms has suggested that a similar gene organisation might be found in *Cupriavidus* species [29]. To identify potential candidates of 3-hydroxypropionate/isobutyrate dehydrogenase genes in *C. necator* H16, the *P. denitrificans* HpdH (H681_18525) and HbdH (H681_13440) proteins were used for primary blastP searches as described in “Methods”. These identified two potential targets: *P. denitrificans* HpdH displayed 60% identity with the product of a gene annotated as choline dehydrogenase (H16_A3663) and located immediately downstream of the *C. necator mmsA*2 gene, whereas HbdH displayed 66% identity with an annotated 3-hydroxyisobutyrate dehydrogenase (H16_B1190) encoded immediately downstream the *C. necator mmsA3* gene. No additional putative *mmsA* homologues could be identified in similar searches using the *P. denitrificans* MmsA protein (H681_13435). These findings suggested that a complete oxidative 3-HP utilisation pathway does indeed exist in *C. necator* (Fig. 2b), but is erroneously presented as incomplete in popular databases such as KEGG pathway [31] due to the misannotation of H16_A3663.

Further interrogation of the regions surrounding the three *C. necator mmsA* genes revealed the presence of additional genes potentially required for (methyl)malonate semialdehyde generation from substrates other than 3-HP. The *aptA* gene (H16_A0272) located immediately upstream of *mmsA1* encodes a putative β-alanine pyruvate transaminase responsible for the conversion of β-alanine to malonate semialdehyde. A gene annotated as *acaD* (H16_B1192) encoding a putative acyl-CoA dehydrogenase is located immediately upstream of *mmsA3*. Downstream of *mmsA3* are the aforementioned *hbdH* (H16_B1190) and also putative genes annotated as encoding 3-hydroxybutyryl-CoA dehydratase (H16_B1189, *crt*) and enoyl-CoA hydratase/isomerase (H16_B1188), all separated by very short intergenic regions.

The above findings suggested the existence of three distinct *mmsA* gene clusters and potentially operons as shown in Fig. 2a. Similar to what has been described for *P. denitrificans*, a divergently transcribed transcriptional regulator gene forms part of each cluster and is located immediately upstream of the respective *mmsA* operon. The existence of three separate operons and their individual composition suggested that they contribute to the degradation of either methylmalonate semialdehyde or malonate semialdehyde generated in different metabolic contexts or from different metabolic precursors. Based on the presence of a putative 3-hydroxypropionate dehydrogenase gene, the *mmsA2* operon was hypothesised to be of primary importance for 3-HP utilisation, whereas the *mmsA3* operon was postulated to have its main role in valine and 3-hydroxyisobutyrate degradation as shown in Additional file 2: Figure S2.

*Cupriavidus necator* also contains a putative malonyl-CoA decarboxylase gene, *mcd* (H16_A2981). As malonyl-CoA may theoretically be formed from malonate semialdehyde or via 3-hydroxypropionyl-CoA/3-oxopropionyl-CoA, this gene was also considered to be of interest.

### Identification of metabolic genes differentially regulated in the presence of 3-HP

Given the existence of three separate *mmsA* operons, a transcriptomic approach was chosen to identify those involved in 3-HP utilisation and to help pinpoint additional, as yet unidentified, contributing genes. For instance, the genes of the 2-methylcitrate pathway, whilst not essential for growth on 3-HP, may still contribute to its conversion. For this, *C. necator* was grown in MM in the presence of either fructose or 3-HP as the sole sources of carbon and energy. Cells were harvested from triplicate cultures at an OD_600_ of 2 (late exponential growth), RNA extracted, processed and subjected to RNA-seq analysis as described in “Methods”. The raw reads were mapped onto the genome sequence of *C. necator* H16 with an overall alignment rate > 93% and analysed using ReadXplorer [32, 33]. The DESeq algorithm was applied to calculate gene-specific fold changes of expression levels [34]. The cohort of genes upregulated on 3-HP included all candidate genes shown in Fig. 2a. This added confidence that the correct gene clusters had been identified. These genes and their relative expression are listed in Additional file 3: Table S1; the entire data sets generated and analysed are available in the ArrayExpress repository under accession number E-MTAB-7701. Interestingly, the *hpdH* and *mmsA2* genes were among the most highly upregulated in the presence of 3-HP (154-fold and 156-fold, respectively; *p*_adj_ = 0.0011) consistent with their proposed primary role in 3-HP degradation. The genes contained within the putative *mmsA1* and *mmsA3* operons were also upregulated (between sevenfold and 18-fold), although not significantly (*p*_adj_ > 0.05), and hence their contribution to 3-HP metabolism remained unconfirmed. By contrast, the genes encoding the methylcitrate pathway presented little changes in transcription and *pct*, encoding propionyl-CoA transferase (H16_A2718), was also not significantly changed, suggesting that they were not required for 3-HP breakdown.

Besides the *mmsA* operons, a number of other metabolic genes displayed differential expression under the two employed conditions (Additional file 3: Table S1; Additional file 4: Figure S3). Among those highly upregulated in the presence of 3-HP were two genes encoding isocitrate lyase isoenzymes (H16_A2211, H16_A2227), in accordance with the proposed breakdown via the oxidative route, allowing the generated acetyl-CoA to enter the tricarboxylic acid cycle (TCA) via the glyoxylate shunt. Interestingly, genes encoding enzymes of the TCA cycle, gluconeogenesis/lower part of the Entner–Doudoroff (ED) pathway and the non-oxidative branch of the pentose phosphate pathway were similarly expressed in 3-HP and fructose grown cells. As might be expected, genes required for fructose uptake, phosphorylation, conversion to glucose-6-phosphate, and first step of the ED pathway were among the most highly downregulated in 3-HP-grown cells (between 78- and 203-fold; *p*_adj_ ≤ 0.0069). Other genes of the upper ED pathway showed relatively small or no changes, none of which were statistically significant. These included the genes for 6-phosphogluconolactonase (H16_B2565, no change), 6-phosphogluconate dehydratase (H16_B2567, no change) and 2-keto-3-deoxy-6-phosphogluconate aldolase (B16_B1213, 3.4-fold downregulated; *p*_adj_ > 0.05) (Additional file 3: Table S1). A second 6-phosphogluconate dehydratase gene, H16_A1178, was strongly downregulated (38-fold), but this change was also not significant under the stringent criteria applied here (*p*_adj_ of 0.0526).

Taken together, the obtained data were consistent with the proposed two-step breakdown of 3-HP via the oxidative route, yielding acetyl-CoA, and highlighted the importance of the *mmsA2* operon.

### Generation of a *C. necator* H16 strain unable to consume 3-HP as a sole source of carbon

**Fig. 3:**
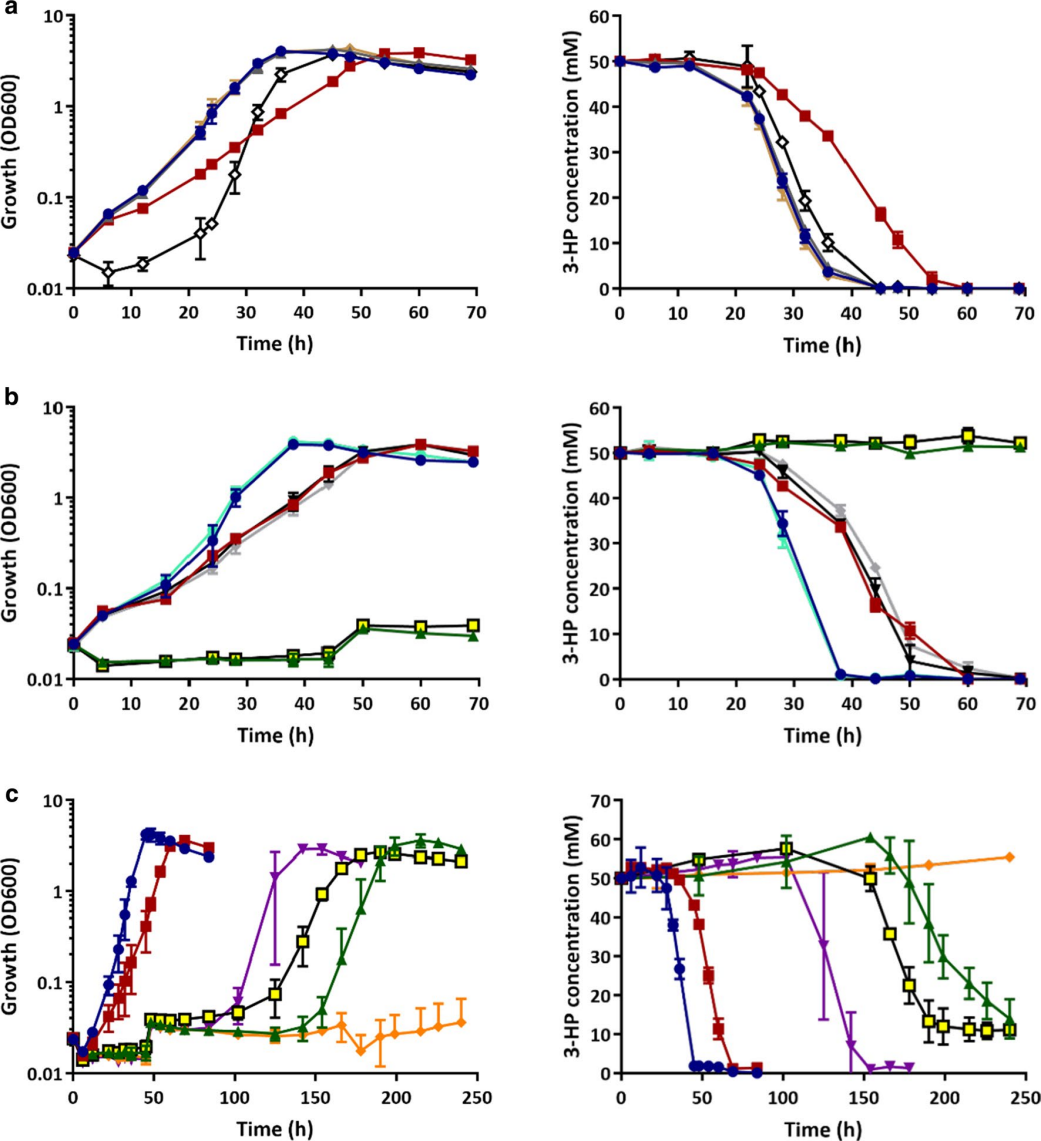
Growth (left) and 3-HP consumption (right) of *C. necator* H16 and generated mutant strains. **a** *C. necator* H16 *mmsA* and *mcd* single knock-out strains. Blue circles represent the H16 wild type; grey triangles, CNCA03 (Δ*mmsA1*); red squares, CNCA04 (Δ*mmsA2*); white diamonds, CNCA05 (Δ*mmsA3*); and brown diamonds, CNCA06 (Δ*mcd*). **b** *C. necator* H16 double deletion mutants. Blue circles represent the H16 wild type; red squares, CNCA04; black inverted triangles, CNCA08 (Δ*mmsA2*Δ*mmsA3*); grey diamonds, CNCA09 (Δ*mmsA2*Δ*mcd*); yellow squares, CNCA10 (Δ*mmsA2*Δ*hpdH*); turquoise circles, CNCA11 (Δ*mmsA3*Δ*hbdH*); and green triangles, CNCA12 (Δ*mmsA2*Δ*mmsA1*). **c** *C. necator* H16 single, double and triple deletion mutants grown over 240 h. Blue circles represent the H16 wild type; red squares, CNCA04; purple inverted triangles, CNCA07 (Δ*hpdh*); yellow squares, CNCA10; green triangles, CNCA12; and orange diamonds, CNCA13 (Δ*mmsA2*Δ*mmsA1*Δ*mmsA3*). Strains were cultivated in MM supplemented with 50 mM 3-HP as the sole source of carbon and energy. Error bars represent the standard deviation of the mean for three independent experiments

The three *mmsA* genes as well as *mcd* were initially selected as targets for gene knockouts to investigate their relative contributions to 3-HP breakdown. The respective genes were inactivated by deletion of their open reading frames (ORFs) and knockouts confirmed by PCR and sequencing as described in “Methods”. The confirmed mutants were designated CNCA03 (Δ*mmsA1*), CNCA04 (Δ*mmsA2*), CNCA05 (Δ*mmsA3*) and CNCA06 (Δ*mcd*), respectively, and grown on 3-HP as the sole sources of carbon and energy to observe whether any of the mutations reduced or even abolished growth. Figure 3a shows growth and 3-HP degradation profiles obtained in the presence of 50 mM 3-HP-MM. All of the mutants eventually grew and completely consumed the provided 3-HP. However, profiles for two of the mutant strains, CNCA04 and CNCA05, differed from that of the wild type. Whilst reaching very similar final optical densities, mutant CNCA04 consumed 3-HP more slowly and, accordingly, grew at a reduced rate. CNCA05 displayed what appeared to be an extended lag phase, resulting in a slight delay in 3-HP consumption. These results, consistent with the above bioinformatics predictions and RNA-seq data, suggested that multiple enzymes contributed to 3-HP utilisation, and in particular those encoded by the *mmsA2* operon. To confirm the relative importance of this operon, the *hpdH* gene was also inactivated and the resulting mutant (CNCA07) compared to a similarly created Δ*hbdH* knockout (CNCA016). In contrast to the latter, deletion of *hpdH* delayed growth and breakdown of 3-HP for about 100 h (Additional file 5: Figure S4), strongly supporting the hypothesis that the encoded 3-HP dehydrogenase together with MmsA2 are the main contributors to 3-HP breakdown in the H16 wild-type strain.

Nevertheless, other enzymes were contributing too, and hence it was decided to generate combinations of the individual mutations generated thus far. CNCA04 was chosen as the starting strain, as the Δ*mmsA2* mutation it contained had already been shown to have a strong effect on 3-HP degradation. The following double mutant strains were generated from CNCA04: CNCA08 (Δ*mmsA2*Δ*mmsA3*), CNCA09 (Δ*mmsA2*Δ*mcd*), CNCA10 (Δ*mmsA2*Δ*hpdH*) and CNCA12 (Δ*mmsA1*Δ*mmsA2*). In addition, strain CNCA11 (Δ*mmsA3*Δ*hbdH*) was generated from the CNCA05 strain. As can be observed in Fig. 3b, the double deletion of *mmsA3* and *hbdH* did not have any effect on growth or degradation of 3-HP as it behaved like the wild-type strain. Furthermore, neither the deletion of *mmsA3* or *mcd* in the Δ*mmsA2* background had an additive effect over Δ*mmsA2* in terms of growth and 3-HP consumption. However, strains CNCA10 (Δ*mmsA2*Δ*hpdH*) and CNCA12 (Δ*mmsA1*Δ*mmsA2*) were unable to grow or utilise 3-HP within the 70 h window shown here and even further until about 120 h and 150 h, respectively. After that time, cells started growing and 3-HP was completely degraded (Fig. 3c). As stated before, a similar behaviour was observed for strain CNCA07, containing a single Δ*hpdH* deletion.

Assuming that this phenomenon was the result of an adaptation, leading to the activation of genes not normally induced under these conditions, the genes already inactivated in CNCA10 and CNCA12 were key candidates for the generation of a strain unable to assimilate 3-HP following additional knock-outs. As HpdH was considered a desirable enzyme to be maintained in a future 3-HP producing strain derivative, only the triple *mmsA* deletion was possible based on the genes identified thus far: Δ*mmsA1*Δ*mmsA2*Δ*mmsA3*. The corresponding strain, designated CNC13, was unable to degrade 3-HP as the sole carbon and energy source within the tested timeframe of up to 250 h (Fig. 3c).

To confirm that combined deletion of the three *mmsA* genes was indeed responsible for lack of 3-HP assimilation rather than second site mutations, it was necessary to complement the individual knockouts in the final mutant strain, CNCA13. As the promoters for these genes had not been experimentally characterised, complementation was carried out by cloning the individual *mmsA* genes into shuttle vector pBBR1MCS-2-P*phaC* [35]. This way, transcription was controlled by the constitutive *phaC* promoter and the different *mmsA* genes could be introduced and expressed separately. Introduction of each *mmsA* gene successfully restored growth of strain CNCA13 on 3-HP as the sole source of carbon and energy (Additional file 6: Figure S5) whereas no discernible growth was observed for the control strain CNCA13 carrying the empty pBBR1MCS-2-P*phaC* vector. Thus, together with the above knockout data, it was confirmed that the products of all three *mmsA* genes contributed to 3-HP breakdown.

### Role of *mmsA3* and *mcd*

**Fig. 4:**
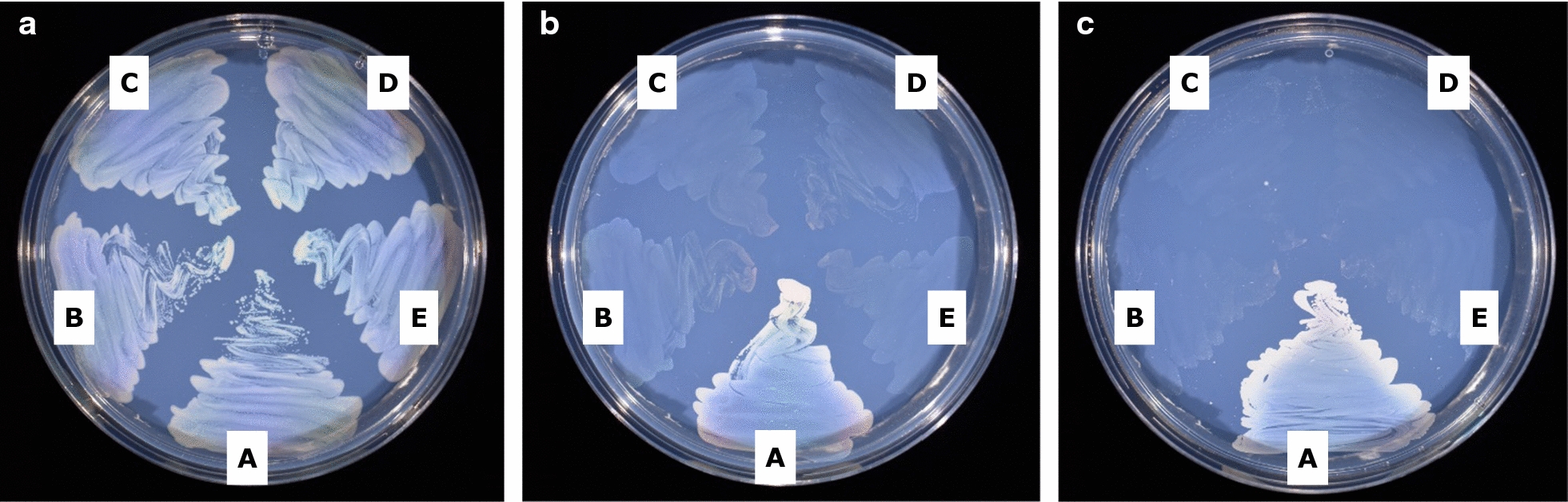
Growth of *C. necator* H16 and its valine degradation deficient mutants. Strains were grown on MM agar plates containing 25 mM fructose (**a**), 30 mM valine (**b**), and 37.5 mM isobutyrate (**c**). A, H16 wild type (positive control); B, CNCA05 (Δ*mmsA3*); C, CNCA16 (Δ*hbdH*); D, CNCA11 (Δ*mmsA3*Δ*hbdH*); and E, CNCA13 (Δ*mmsA1*Δ*mmsA2*Δ*mmsA3*). Agar plates were incubated at 30 °C for 5 days

Having constructed various Δ*mmsA* operon and Δ*mcd* mutants, it was of interest to better understand the primary roles of the encoded gene products and their individual contributions to C_3_ metabolism. Located close to *mcd* is a gene annotated to encode a putative malonyl-CoA synthetase (H16_A2978). It therefore seemed likely that both genes were required for growth on malonic acid, a compound accumulated to a high level by certain plants [36]. However, when the H16 parent and its CNCA06 (Δ*mcd*) derivative were cultivated in MM with malonate as the sole source of carbon and energy (50 mM, 25 mM, 12.5 mM), no growth was observed for either strain. Thus, the role of the *mcd* gene product in *C. necator* metabolism remained uncertain. For *mmsA3*, based on the proposed function of the other genes within the operon, a role in valine degradation had been hypothesised (Additional file 2: Figure S2). This was tested by comparing the growth of the Δ*mmsA3* and Δ*hbdH* mutants (CNCA05, CNCA11, CNCA13 and CNCA16) with that of the H16 parent strain in the presence of valine as the sole source of carbon and energy. Isobutyrate was also tested, as the derived isobutyryl-CoA is another intermediate in the valine degradation pathway. As can be seen in Fig. 4, in contrast to the H16 parent strain, all tested mutants failed to grow on these substrates in support of the formulated hypothesis. Further confirmation was obtained through genetic complementation. Introducing complementation vectors pBBR1MCS-2-P_*phaC*_-*mmsA3* and pMTL71301-P_*acaD*_-*hbdH* into the CNCA05 (Δ*mmsA3*) and CNCA16 (Δ*hbdH*) mutant, respectively, restored growth on valine, in contrast to the empty plasmid controls (Additional file 7: Figure S6). The same result was obtained for the CNCA13 (Δ*mmsA1*Δ*mmsA2*Δ*mmsA3*) triple mutant when containing pBBR1MCS-2-P_*phaC*_-*mmsA3*. Interestingly, complementation of the latter with either *mmsA1* or *mmsA2* also restored growth on valine (Additional file 7: Figure S6), suggesting that all three *mmsA* gene products recognised both methylmalonate semialdehyde and malonate semialdehyde as substrates. Oxidative decarboxylation of methylmalonate semialdehyde by MmsA enzymes as part of the valine degradation pathway yields propionyl-CoA (Additional file 2: Figure S2). Hence the generated methylcitrate pathway mutant, CNCA15, was also tested and, as expected, shown to be unable to grow on valine (Additional file 8: Figure S7).

### Degradation of 3-HP in *mmsA* mutant strains under mixotrophic conditions

The Δ*mmsA1*Δ*mmsA2*Δ*mmsA3* triple deletion had generated a strain unable to utilise 3-HP as the sole source of carbon and energy. However, it remained unclear whether this strain could still co-metabolise 3-HP alongside fructose or other carbon sources. In order to investigate this possibility and see the effect of each individual *mmsA* deletion step as well, the different strains (wild type, CNCA04, CNCA12 and CNCA13) were incubated in MM in the presence of 25 mM fructose and 50 mM 3-HP.

**Fig. 5:**
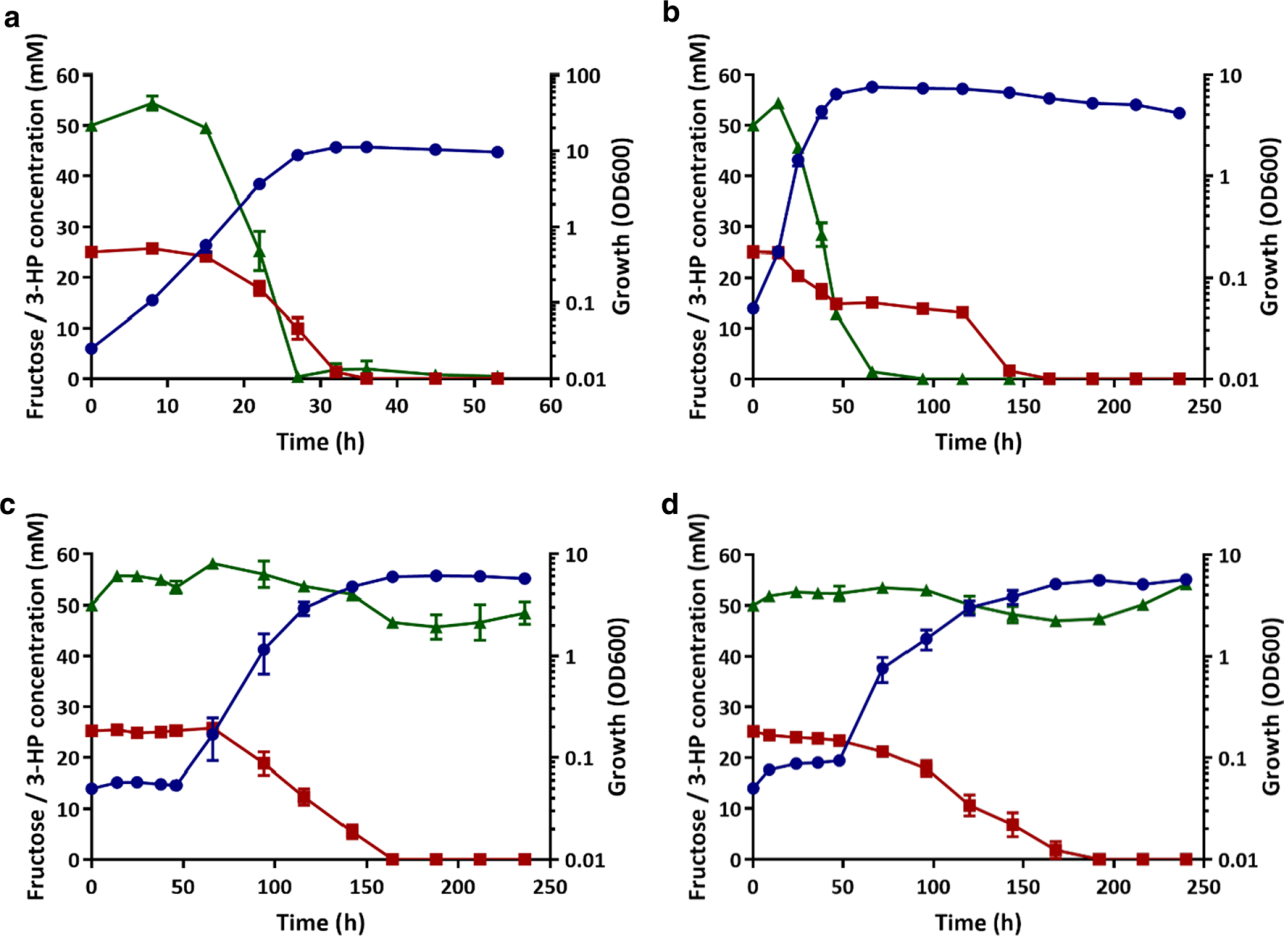
Co-consumption of fructose and 3-HP by *C. necator* H16 and Δ*mmsA* mutant strains. **a** *C. necator* H16 wild type; **b** CNCA04 (Δ*mmsA2*); **c** CNCA12 (Δ*mmsA1*Δ*mmsA2*); **d** CNCA13 (Δ*mmsA1*Δ*mmsA2*Δ*mmsA3*). Strains were cultivated in MM in the presence of 25 mM fructose and 50 mM 3-HP. Blue circles, OD_600_; red squares, fructose; green triangles, 3-HP. Error bars represent the standard deviation of the mean for three independent experiments

As can be seen in Fig. 5a, *C. necator* H16 wild type was able to co-metabolise both substrates without any clear preference, although 3-HP appeared to be consumed more rapidly and was therefore depleted earlier than fructose. A similar result was observed for the Δ*mmsA2* mutant, CNCA04 (Fig. 5b), although, as observed when cultivated on 3-HP only, it grew more slowly and took twice as long to consume 3-HP (about 70 h), with fructose only being depleted after 150 h, long after growth had ceased. Similarly, the double deletion strain CNCA12 (Δ*mmsA1*Δ*mmsA2*; Fig. 5c) and the triple mutant strain CNCA13 (Δ*mmsA1*Δ*mmsA2*Δ*mmsA3*; Fig. 5d), whilst not metabolising the provided 3-HP over the entire course of the experiment (240 h) displayed drastically increased lag phases. An initial growth delay of approximately 50 h was observed for both strains in comparison to the wild-type strain and fructose was consumed comparatively slowly with depletion occurring more than 150 h after inoculation. This may have been the result of regulatory control, leading to reduced expression of fructose utilisation genes due to the continued presence of 3-HP or absence of its breakdown intermediates.

**Fig. 6:**
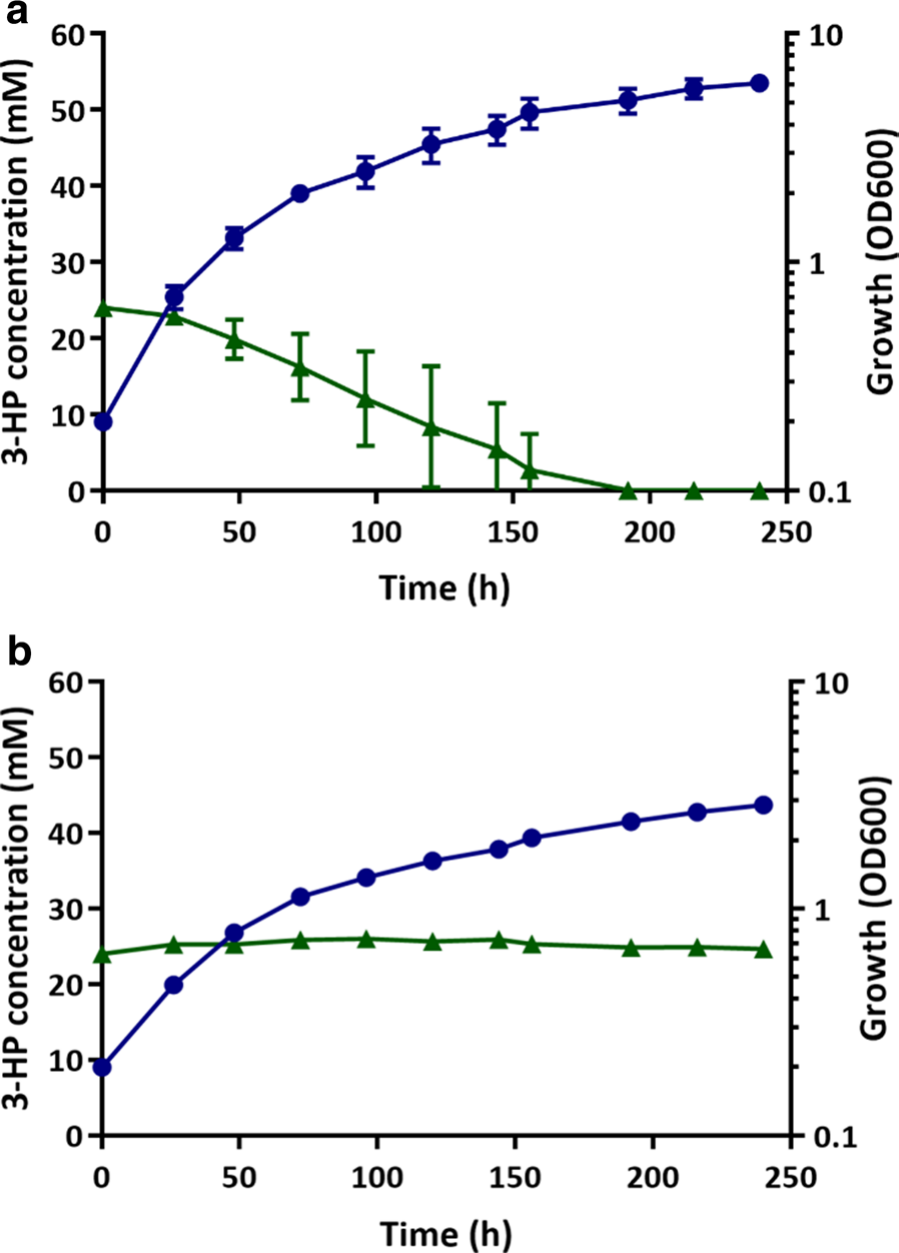
3-HP consumption under mixotrophic conditions. **a** *C. necator* H16 wild type, **b** CNCA13 (Δ*mmsA1*Δ*mmsA2*Δ*mmsA3*) mutant. The strains were cultivated in 240 mL serum bottles containing 24 mL of MM supplemented with 24 mM 3-HP and an atmosphere of hydrogen, carbon dioxide and air in a ratio of 8:1:1 (v/v/v). Blue circles, OD_600_; green triangles, 3-HP. Error bars represent the standard deviation of the mean for three independent experiments

The ultimate aim of this study was to engineer *C. necator* strains unable to degrade 3-HP when using CO_2_ and H_2_ as carbon and energy sources. The H16 wild type and CNCA13 strains were hence tested for 3-HP degradation when cultivated under mixotrophic conditions. For this, both strains were grown in serum bottles with butyl-rubber stoppers containing 24 mM 3-HP in MM and a gas mixture of H_2_, CO_2_ and air for a period of 240 h (see “Methods” for details). Whilst 3-HP was utilised by the wild type cultures under these conditions and completely depleted after 190 h, there was no discernable consumption by the triple *mmsA* deletion strain, CNCA13, which nevertheless grew on the provided CO_2_/H_2_ mixture (Fig. 6).

## Discussion

Several microorganisms are known to degrade and grow on 3-HP, including *R. sphaeroides, P. denitrificans, Methylobacterium extorquens* and several other proteobacteria [15, 29, 37]. *C. necator* has previously been shown to utilise 3-HP to generate PHA co-polymers consisting of 3-HP and 3-HB [38]. Here, we demonstrate that 3-HP is co-metabolised by the H16 strain in the presence of other carbon sources and can also serve as the sole source of carbon and energy.

*Cupriavidus necator* is a promising platform organism for the autotrophic production of 3-HP. Hence, from a metabolic engineering perspective, the observed degradation was a highly undesirable trait that required elimination. A prerequisite for this was the identification of contributing pathways and their encoding genes.

Two principle pathways for 3-HP breakdown have been described, a reductive and an oxidative route. Reductive breakdown as described for *R. sphaeroides* requires activation to 3-HP-CoA, reduction to propionyl-CoA and further conversion of the latter via the methylcitrate pathway, yielding pyruvate and succinate [15, 25]. The oxidative route as present in, e.g. *P. denitrificans* is less carbon-efficient but much shorter: Oxidation of 3-HP yields malonate semialdehyde which is then decarboxylated to acetyl-CoA [17, 29].

Putative genes for both pathways are present in the H16 strain, but contrary to previous suggestions [24], 3-HP appears to be utilised exclusively via the oxidative route, at least under conditions employed in this study: inactivation of methylcitrate pathway genes did not affect growth on 3-HP, whilst combined inactivation of three malonate semialdehyde dehydrogenases completely abolished growth and 3-HP breakdown. Thus, *C. necator* oxidises 3-HP to acetyl-CoA, whereas propionate is metabolised via a functional methylcitrate pathway [25], despite presence of a broad-specificity CoA transferase, Pct, capable of converting 3-HP to 3-HP-CoA [21, 22].

*Cupriavidus necator* is metabolically highly versatile [39, 40] and known to employ multiple isoenzymes for key reactions in many of its metabolic pathways. Indeed, three *mmsA* genes and at least two candidates for 3-HP dehydrogenation are present in the H16 genome and form part of three different predicted operons. Presumably these operons are serving different primary functions and therefore guidance was sought through comparative RNA-seq analysis as to which may contribute to 3-HP degradation. However, whilst the *mmsA2* operon was amongst the most highly up-regulated genes, transcript levels for the other two operons were also increased in the presence of 3-HP, suggesting that they also contributed to its consumption. This was experimentally confirmed through a series of single, double and triple knockout mutants. The generated final *mmsA* triple knockout strain, CNCA13 showed no discernible consumption of 3-HP, even in the presence of organic (fructose) or inorganic (CO_2_/H_2_) co-substrates and an extended incubation period of 10 days, hence realising the main aim of this study, the engineering of a strain no longer capable of utilising 3-HP as a carbon and energy source.

Among the single knockouts, *hpdH* inactivation was of particular interest due to its strong phenotype. Inactivation of the gene delayed growth on 3-HP for more than 100 h, thus underlining the key role of the *mmsA2* operon in 3-HP conversion. Although *hpdH* was a potential candidate for 3-HP knockout strain construction, this option was not pursued. Presence of the gene was considered desirable as it might bolster the organism’s capacity to convert potentially toxic [41] malonate semialdehyde to 3-HP in the last step of both malonyl-CoA and β-alanine production pathways [42–44]. However, inactivation of the native gene could still be an option for future engineering work. For instance, it might be preferable to replace it with a more suitable alternative, depending on the desired cofactor preference for this reaction (i.e. NADPH vs NADH) or the required kinetic parameters.

Given that inactivation of all three *mmsA* genes was required to abolish 3-HP consumption, a better understanding of the encoding operons and their individual roles in C_3_ metabolism was sought as part of this study to ensure they did not affect other important functions. *mcd* was included in this analysis as malonate and malonyl-CoA have been reported to accumulate when 3-HP-grown cultures of *P. denitrificans* were treated with the acid dehydrogenase inhibitor 4-hydroxy mercuric benzoic acid [26]. Due to the presence of a putative malonyl-CoA synthetase gene close by, *mcd* was hypothesised to be required for malonate breakdown but this could not be confirmed experimentally as both Δ*mcd* mutant and H16 parent strain were unable to grow on malonate under the chosen conditions. However, the gene does not appear to be involved in 3-HP breakdown as inactivation of all three *mmsA* homologues was shown to be sufficient to block degradation. The *mmsA1* operon encodes a second gene, *aptA*, annotated as β-alanine-pyruvate transaminase. Hence a primary role in the utilisation of β-alanine generating substrates is proposed. Indeed, *C. necator* H16 has been reported to degrade and grow on β-alanine and carnosine, but not uracil [45, 46]. As detailed above, the *mmsA2* operon was highly upregulated in the presence of 3-HP, in agreement with previous reporter studies [47], and also encodes the aforementioned 3-hydroxypropionate dehydrogenase (HpdH) required for efficient 3-HP breakdown. Hence its physiological role as confirmed here is in the degradation of 3-HP and, potentially, 3-HP generating substrates (one of which might be myo-inositol as observed in *Lactobacillus casei* [48]). As outlined in Additional file 2: Figure S2, the genes contained within the putative *mmsA3* operon were postulated to have their main role in valine and 3-hydroxyisobutyrate degradation based on their predicted functions and this was confirmed experimentally for both Δ*mmsA3* and Δ*hbdH* knockouts. The respective mutants were unable to grow on valine and isobutyrate within 5 days. Based on the above, it can be assumed that under normal physiological conditions MmsA1 and MmsA2 act primarily on malonate semialdehyde whereas the physiological substrate for MmsA3 is methylmalonate semialdehyde. However, despite their separate physiological roles, the enzymes’ substrate specificities appear to be sufficiently broad to enable conversion of both substrates, as illustrated by ability of the different CACA13 complementation strains to grown on both valine and 3-HP. This is in agreement with the literature. Whilst a high substrate specificity has been reported for a malonate semialdehyde dehydrogenase isolated from *Pseudomonas* sp. strain AAC [49], other enzymes are known to accept both substrates [50–52].

It is reasonable to assume that the three *C. necator mmsA* operons as well as *mcd* are controlled by dedicated transcriptional regulators encoded immediately upstream of each operon, as has been observed for many bacteria and specifically for the *mmsA* operons in *P. denitrificans* [29, 47, 53]. Control of *mmsA2* by the upstream encoded LysR-type regulator appears likely but requires further confirmation as the system was not responsive to 3-HP when expressed heterologously in *E. coli* [47]. Similar control of the *mmsA1* and *mmsA3* operons via the upstream encoded MocR and AraC-type regulators may also explain the long delay in 3-HP consumption observed for the Δ*mmsA1*Δ*mmsA2* (CNCA12) and Δ*mmsA2*Δ*hpdH* (CNCA10) double mutants as well as the long lag phase observed for the Δ*mmsA1*Δ*mmsA2*Δ*mmsA3* (CNCA13) mutant when grown on fructose together with 3-HP. Whilst our H16 wild type data suggest that both carbon sources can be used in parallel, at least at some stages of growth, 3-HP appears to be consumed more rapidly. It was thus possible that the continuingly high levels of 3-HP in our Δ*mmsA1*Δ*mmsA2*Δ*mmsA3* (CNCA13) mutant cultures prevented or delayed sufficient induction of fructose utilisation genes.

Comparative RNA-seq analysis of 3-HP and fructose-grown cells was primarily performed to obtain guidance as to which of the three *mmsA* operons and potentially other genes were involved in 3-HP utilisation. In addition, the obtained data yielded some interesting insights into the transcriptional changes of genes associated with central carbon metabolism (Additional file 4: Figure S3). These changes were consistent with the implemented change in carbon source and oxidative conversion of 3-HP to acetyl-CoA. When grown on 3-HP, as might be expected, genes required for fructose uptake and conversion via the upper part of the ED pathway were strongly downregulated. Among those highly upregulated in the presence of 3-HP were two genes encoding isoenzymes of isocitrate lyase (*aceA1,* H16_A2211 and *aceA2*, H16_A2227). The same genes were previously shown to be upregulated during growth on trioleate [7], the fatty acid components of which are metabolised via β-oxidation and, like acetate, enter the tricarboxylic acid cycle via acetyl-CoA. In the absence of other significant anaplerotic fluxes into the TCA, net assimilation of carbon is facilitated by the glyoxylate shunt, a pathway that circumvents the decarboxylation reactions of the TCA cycle [7, 54], with isocitrate lyase catalysing the first reaction of said pathway. Hence, increased expression of the isocitrate lyase genes is in accordance with the proposed oxidative 3-HP assimilation pathway which yields acetyl-CoA. The *aceB1* gene encoding malate synthase, the second enzyme of the glyoxylate bypass, was not upregulated, in contrast to what had been reported for growth on trioleate [7]. However, AceB1 only accounts for part of the cell’s malate synthase activity and the presence of a second as yet unidentified enzyme has thus been proposed [54].

## Conclusions

This study engineered a *C. necator* strain unable to consume 3-HP under all conditions tested and elucidated the primary roles of the three different *mmsA* operons present in the organism. These roles are linked to the degradation of β-alanine, 3-HP and valine, respectively. Nevertheless, inactivation of all three methylmalonate semialdehyde dehydrogenases was required to completely abolish 3-HP breakdown. The generated CNCA13 strain, *C. necator* H16 Δ*mmsA2*Δ*mmsA1*Δ*mmsA3*, is an ideal chassis for the future biosynthesis of 3-HP.

## Methods

### Bacterial strains, medium and growth conditions

All species and strains used in this study are listed in Additional file 9: Table S2. Standard lysogeny broth (LB) was used for general maintenance of *E. coli* and *C. necator* strains. Low-salt-LB (LSLB)-MOPS medium [55] was used when growing *C. necator* H16 as recipient in conjugative procedures and Hanahan’s Broth (SOB Medium—H8032, Sigma-Aldrich) for the preparation of *C. necator* H16 competent cells. Chemically defined medium (MM) [56] including modified trace element solution SL7 [57] was used for *C. necator* H16 growth assays and was supplemented with either 25 mM d-fructose or 50 mM 3-HP unless otherwise stated. For mixotrophic cultivation, the cultures were grown in 240 mL serum bottles with hydrogen, carbon dioxide and air in a ratio of 8:1:1 (v/v/v) [1] containing 24 mL MM supplemented with 24 mM 3-HP (purchased from Apollo Scientific as 30% aqueous solution). 48 mL of air was added to the bottles every 24 h to ensure oxygen availability. If appropriate, antibiotics were added to the medium at the following concentrations: 50 µg/µL chloramphenicol or 12.5 µg/µL tetracycline. Unless stated otherwise, *E. coli* and *C. necator* H16 strains were grown aerobically in a shaking incubator (Thermo Scientific™ MaxQ™ 8000 Incubated Stackable Shaker) at 37 and 30 °C, respectively, with shaking at 200 rpm.

### Plasmid isolation, PCR, cloning and transformation

Plasmid DNA was isolated using the Monarch Plasmid Miniprep Kit (New England BioLabs Inc. (NEB)). Genomic DNA extractions were performed using the GenElute Bacterial Genomic DNA Kit (Sigma-Aldrich). Unmodified oligonucleotide primers from Eurofins MWG were used for PCR amplification. PCRs were performed using DreamTaq PCR Master Mix (×2) (Thermofisher Scientific) and Q5 High-Fidelity 2× Master Mix (NEB) following the manufacturers’ protocols. DreamTaq PCR Master Mix was used for screening of clones by colony PCR whereas Q5 polymerase was used for QuickChange site-directed mutagenesis and amplification of sequences for cloning. Restriction enzymes were purchased from NEB and “FastDigest” enzymes from Fermentas (Thermofisher Scientific). Ligation reactions were performed using T4 DNA ligase (NEB). The Monarch DNA Gel Extraction Kit (NEB) was used to extract gel purified DNA for subsequent cloning. NEBuilder HiFi DNA assembly mastermix was purchased from NEB and used for assembly of vectors. Chemical competent *E. coli* were prepared and transformed by heat shock as previously described [58]. Plasmid constructs were introduced into *Cupriavidus* via electroporation [59] or conjugation, following the procedure described by Lenz et al. [55]. Plasmids used or generated in this study are listed in Additional file 10: Table S3; all oligonucleotides are listed in Additional file 11: Table S4.

### Construction of deletion and complementation vectors

Derivatives of suicide plasmid pLO3 were used to carry out deletions in *C. necator* H16. The pLO3 backbone contains a tetracycline resistance marker and the *sacB* gene, allowing for counter-selection in the presence of sucrose [60]. Constructed pLO3-derived vectors consisted of two homology arms of ~ 1000 bp each, flanking the gene of interest upstream and downstream of the start and stop codon, respectively, and fused together using the HiFi DNA Assembly Kit (NEB) following manufacturer’s protocol. Briefly, oligonucleotide primers containing homology regions of the vector and the gene of interest were designed using NEBuilder^®^ Assembly Tool. Homology arms were amplified by PCR, analysed by agarose gel electrophoresis and extracted from the gel as described above. pLO3 vector was digested with SacI/XbaI and recovered following the same procedure as for the homology arms. For final assembly, digested vector and purified homology arms were mixed in a final volume of 5 µL and, following addition of 5 µL of 2× NEBuilder HiFi DNA Assembly Master Mix, incubated at 50 °C for 1 h. The resulting assembly reaction was used to transform *E. coli* DH5α chemically competent cells. Candidate plasmids were isolated, verified by sequencing, and electroporated into the conjugation donor strain *E. coli* S17-1 λpir. Precise positions of the cloned flanking regions can be derived from the oligonucleotide sequences given in Additional file 11: Table S4.

Genetic complementation of *mmsA* mutants was carried out by cloning the targeted genes into pBBR1MCS-2-PphaC [35]. This way, the transcription was controlled by the constitutive *phaC* promoter and constructs could be expressed individually in strain CNCA13. The Δ*hbdH* mutant was genetically complemented by cloning the targeted gene into pMTL71301 (Dr Muhammad Ehsaan, University of Nottingham, unpublished) under the control of the native P_*acaD*_ promoter (i.e. the promotor upstream of the *mmsA3* operon). The oligonucleotides used to amplify the complementation fragments are listed in Additional file 11: Table S4.

### Cell cultivation and sampling for transcriptome analyses

Three single colonies of *C. necator* H16 grown on solid LB medium were spread on solid minimal media containing fructose (25 mM). Inoculated minimal media plates were incubated for 2 days at 30 °C. From these plates, precultures were prepared in 50 mL PE tubes containing 10 mL MM with either fructose (25 mM) or 3-HP (50 mM). Single *C. necator* colonies were used for inoculation and precultures were incubated at 30 °C and 180 rpm in a shaking incubator. Precultures with fructose as sole carbon source were incubated for 1 day, whereas precultures with 3-HP were incubated for 2 days. Main cultures were performed in triplicate in 25 mL of the same medium in 250 mL baffled shake flasks and were inoculated from the respective precultures to a starting OD_600_ of 0.025. Flasks were incubated at 30 °C and 180 rpm in a shaking incubator and growth was monitored by measuring optical density at 600 nm. Samples for RNA isolation were taken during late exponential growth (OD_600_ ~ 2) by rapid centrifugation (~ 30 s, max. speed) in 1.5 mL reaction tubes and quenching of cell pellets in liquid nitrogen. Pellets were stored at − 80 °C prior to RNA isolation.

### RNA isolation and transcriptome analysis via RNA-seq

Cell pellets were disrupted using the FastRNA PRO BLUE KIT (MP Biomedicals). On ice, two cell pellets of each culture were resuspended in 500 µL of RNApro™ solution (containing Trizol) and combined in a single 2 mL screw-cap tube with Lysing Matrix B. Cell pellets were disrupted in a tissue homogeniser (Precellys 24, Peglab) at 6000 rpm for 40 s. After centrifugation at 12,000×*g* the supernatant was transferred to a new microcentrifuge tube and incubated at room temperature for 5 min, before RNA was extracted from the lysate using a DirectZol RNA Miniprep kit (Zymo Research), according to the manufacturer’s protocol. After elution of RNA in a total volume of 50 µL RNAse-free water, the samples were treated with 2.6 U of Ambion TURBO DNAse in a total reaction volume of 60 µL for 1 h at room temperature. The RNA was purified again using the Clean & Concentrator kit (Zymo Research) according to the manufacturer instructions. The absence of DNA in the total RNA preparation was confirmed by PCR using two individual primer pairs (DNA_Cn1/2, Additional file 11: Table S4) and genomic *C. necator* H16 DNA as a positive control.

The depletion of rRNA, cDNA library preparation and sequencing were done as described in Wittchen et al. [61]. However, in this case equal RNA amounts from the three replicates of each condition were pooled, and 2 µg RNA of each pool were used for the creation of a single whole transcriptome cDNA library. The resulting paired-end reads from the sequencing of the two libraries from *C. necator* grown with fructose and 3-HP as sole carbon sources were trimmed for low quality bases from both ends using Trimmomatic v0.35 [62] and mapped to the reference genome (NC008313.1, NC008314.1, NC005241.1) using bowtie2 v2.2.7 [63] with default settings for paired-end read mapping. The resulting SAM files were converted to BAM files using SAMtools [64]. ReadXplorer v.2.2.3 was used for visualisation and further analysis [32, 33]. For differential gene expression analysis the inbuilt DESeq algorithm [34] was applied with default settings (cutoffs for which genes were considered as differentially transcribed). The adjusted *p* value (*p*_adj_) calculated by the DESeq algorithm using Benjamini–Hochberg correction was accepted as a measure of significance. The full data has been deposited at ArrayExpress (https://www.ebi.ac.uk/arrayexpress/) and is accessible under accession number E-MTAB-7701.

### HPLC detection of fructose and 3-HP

Culture samples were centrifuged at 14,000 rpm for 2 min and the supernatants used for product analysis. Diluent solution was prepared using mobile phase (0.005 M H_2_SO_4_) supplemented with 50 mM valeric acid (Sigma-Aldrich) acting as an internal standard. Also, an appropriate linear range of standard solutions using 3-HP (Apollo Scientific) covering the range of concentrations expected was prepared in the medium used for the corresponding experiment. All standards, samples and blanks were passed through 0.2 µm syringe filters, and 150 µL of the filtered solution mixed with 150 µL of diluent solution, vortexed thoroughly and added into HPLC vials containing inserts and closed with split caps. Samples were run at a flow rate of 0.5 mL min^−1^ at 35 °C in 0.005 M H_2_SO_4_ mobile phase for 30 min on the Dionex UltiMate 3000 HPLC system (ThermoFisher Scientific) using a Rezex ROA-Organic Acid H + (8%) 150 mm × 4.6 mm × 8 µm column (Phenomenex) and a Diode Array detector (UV–VIS 210 nm). An Aminex HPX-87H 300 mm × 7.8 mm × 9 µm column (Bio-Rad) was used when a better separation of peaks was needed and samples were run for 55 min.

### Sequence databases and BLAST analysis

The databases at the National Center for Biotechnology Information (NCBI) were used to identify and access nucleotide sequences and peptide sequences of genes of interest [65]. The Kyoto Encyclopedia of Genes and Genomes (KEGG) was used to access potential pathways and annotated genes [31]. BLAST software, available on the NCBI website, was used to analyse sequences and identification of similarity. Nucleotide BLAST (blastn) optimised for highly similar sequences (megablast) was used to identify similar or identical DNA sequences; blastp (protein–protein blast) was used to identify putative protein homologs.

## Additional files

**Additional file 1: Figure S1.** Growth of *C. necator* H16 wild type and CNCA15 (Δ*prpRBCMD*) mutant strains on 3-HP and propionate. Strains were cultivated in MM supplemented with 50 mM 3-HP (a) or propionate (b) as the sole source of carbon and energy. Blue circles represent H16 wild type, red squares the CNCA15 (Δ*prpRBCMD*) mutant. Error bars represent the standard deviation of the mean for three independent experiments.

**Additional file 2: Figure S2.** Proposed valine degradation pathway in *C. necator* H16. (a) Valine degradation pathway with associated *C. necator* enzymes for each reaction. (b) Putative *C. necator* valine degradation operon and upstream regulatory gene, together with associated locus tags. Genes are proposed to encode the following enzymes: AraC family transcriptional regulator (*araC*, H16_B1193), branched-chain acyl-CoA dehydrogenase (*acaD,* HB161192), (methyl)malonate semialdehyde dehydrogenase (*mmsA3*, H16_B1191), 3-hydroxyisobutyrate dehydrogenase (*hbdH*, H16_B1190), enoyl-CoA dehydratase (*crt*, H16_B1189) and 3-hydroxyisobutyryl-CoA hydrolase (*hibH*; note: the gene is currently annotated to encode an enoyl-CoA hydratase/isomerase, H16_B1188). Glu, glutamate; α-KG, α-ketoglutarate. The first two steps of valine degradation are carried out by branched-chain amino acid aminotransferase and 3-methyl-2-oxobutanoate dehydrogenase (2-oxoisovalerate dehydrogenase), respectively; their encoding genes are not part of this operon. Blue colours indicate reactions and genes associated with the proposed *mmsA3* operon. Sizes of genes and intergenic regions not drawn to scale.

**Additional file 3: Table S1.** Genes of interest differentially expressed on 3-HP compared to fructose.

**Additional file 4: Figure S3.** Differential expression of key metabolic genes in *C. necator* H16 during exponential phase growth on 3-HP and fructose, respectively. Red and green boxes indicate genes significantly up- and downregulated respectively, when cells were grown on 3-HP (*p*_adj_ <0.05). Grey boxes indicate no statistically significant differential regulation. Locus tags are given to enable unambiguous gene identification. Abbreviations: F6P, fructose-6-phosphate; G6P, glucose-6-phosphate; 6PGL, 6-phosphogluconolactone; 6PG, 6-phosphogluconate; KDPG, 2-keto-3-deoxy-6-phosphogluconate; FBP, fructose-1,6-bisphosphate; GAP, glyceraldehyde-3-phosphate, DHAP, dihydroxyacetone phosphate; S7P, sedoheptulose-7-phosphate; R5P, ribose-5-phosphate; X5P, xylulose-5-phosphate; E4P, erythrose-4-phosphate; 1,3PG, 1,3-phosphoglycerate; 3PG, 3-phosphoglycerate; 2PG, 2-phosphoglycerate; PEP, phosphoenolpyruvate; PYR, pyruvate; AcCoA, acetyl-CoA, CIT, citrate; ISOCIT, isocitrate, AKG, α-ketoglutarate; SUCC, succinate; FUM, fumarate; MAL, malate, OAA, oxaloacetate; GLYOX, glyoxylate; 3HP, 3-hydroxypropionate; MSA, malonate semialdehyde; 3HP-CoA, 3-hydroxypropionyl-CoA; AcrCoA, acryloyl-CoA; 3Prp-CoA, propionyl-CoA; MeCIT, methylcitrate; MeISOCIT, methylisocitrate.

**Additional file 5: Figure S4.** Growth (a) and 3-HP consumption (b) of *C. necator* strains CNCA07 (Δ*hpdH*) and CNCA16 (Δ*hbdH*). Strains H16, CNCA07 (Δ*hpdH*) and CNCA16 (Δ*hbdH*) were cultivated in MM supplemented with 50 mM 3-HP as the sole source of carbon and energy. Blue circles, H16 wild type; inverted purple triangles, CNCA07; pink diamonds, CNCA16. Error bars represent the standard deviation of the mean for three independent experiments.

**Additional file 6: Figure S5.** Cell growth (a) and 3-HP consumption (b) of *C. necator* CNCA13 complementation strains. The different CNCA13 (Δ*mmsA1*Δ*mmsA2*Δ*mmsA3*) complementation strains carrying plasmids pBBR1MCS-2-P_*phaC*_-*mmsA1* (green triangles), pBBR1MCS-2-P_*phaC*_-*mmsA2* (inverted purple triangles), and pBBR1MCS-2-P_*phaC*_-*mmsA3* (orange diamonds), respectively, were cultivated in MM supplemented with 50 mM 3-HP as the sole source of carbon and energy. Controls included H16 wild type (blue circles) and CNCA13 (brown squares) carrying the empty pBBR1MCS-2-P_*phaC*_ vector. Error bars indicate the standard deviation of the mean for three independent experiments.

**Additional file 7: Figure S6.** Genetic complementation of CNCA16 (*Δhbdh*), CNCA05 (Δ*mmsA*3) and CNCA13 (*ΔmmsA1ΔmmsA2ΔmmsA3*) mutant strains restores growth on valine. (a) Growth of *C. necator* H16 wild type, CNCA16 (Δ*hbdH*) mutant and complemented CNCA16 (Δ*hbdH*) mutant. Strain H16 (A) and the CNCA16 mutant control (B) contained empty pMTL71301 vector, the complemented CNCA16 mutant (C) contained plasmid pMTL71301-P_*acaD*_-*hbdh.* (b) Growth of *C. necator* H16 wild type, CNCA05 (Δ*mmsA*3) mutant and complemented CNCA05 (Δ*mmsA*3) mutant. Strain H16 (A) and the CNCA05 mutant control (B) contained empty pBBR1MCS-2-P_*phaC*_-*eyfp* vector, the complemented CNCA05 mutant (C) contained plasmid pBBR1MCS-2-P_*phaC*_-*mmsA3.* (c) Growth of *C. necator* H16 CNCA13 (Δ*mmsA1*Δ*mmsA2*Δ*mmsA3*) complemented with either *mmsA1*, *mmsA2* and *mmsA3* genes. Strain H16 (A) and the CNCA13 mutant control (B) contained empty pBBR1MCS-2-P_*phaC*_-*1*-*eyfp* vector, whereas the complemented CNCA13 mutants contained pBBR1MCS-2-P_*phaC*_-*mmsA1* (C), pBBR1MCS-2-P_*phaC*_-*mmsA2* (D) and pBBR1MCS-2-P_*phaC*_-*mmsA3* (E), respectively. All strains were grown on MM agar plates containing 25 mM fructose (i) and 30 mM valine (ii). Agar plates were incubated at 30 °C for 5 days.

**Additional file 8: Figure S7.** Growth of *C. necator* H16 and CNCA15 (Δ*prpRBCMD*) mutant strains on fructose and valine. Strains H16 (A) and CNCA15 (B) were grown on MM agar plates containing 25 mM fructose (a, left panel) and 30 mM valine (b, right panel). Agar plates were incubated at 30 °C for 5 days.

**Additional file 9: Table S2.** Strains used and generated in this study.

**Additional file 10: Table S3.** Plasmids used and generated in this study.

**Additional file 11: Table S4.** Primers used in this study.

## Abbreviations

3-HB: 3-hydroxybutyric acid
3-HP: 3-hydroxypropionic acid
ED: Entner–Doudoroff pathway
F-MM: minimal medium containing 0.4% fructose
h: hour
*hbdH*: 3-hydroxyisobutyrate dehydrogenase gene
*hpdH*: 3-hydroxypropionate dehydrogenase gene
LB: lysogeny broth
LSLB: low-salt lysogeny broth
*mcd*: malonyl-CoA decarboxylase gene
MM: minimal medium
*mmsA*: (methyl)malonate semialdehyde dehydrogenase gene
OD: optical density
ORF: open reading frame
p_adj_: adjusted p value
Pct: propionate CoA-transferase
PHA: polyhydroxyalkanoate
PHB: polyhydroxybutyrate
TCA: tricarboxylic acid cycle
TDP: 3,3′-thiodipropionic acid

## Availability of data and materials

The full transcriptomic dataset generated and analysed during the current study is available in the ArrayExpress repository (https://www.ebi.ac.uk/arrayexpress/) and is accessible under E-MTAB-7701.
