# Supplementary material for: The genetic basis of 3-hydroxypropanoate metabolism in Cupriavidus necator H16
Source: Biotechnol Biofuels. 2019 Jun 17;12:150. doi: 10.1186/s13068-019-1489-5 (PMC6572756; doi:10.1186/s13068-019-1489-5)
Supplement: Supplementary file 1 — Additional file 1: Figure S1. Growth of C. necator H16 wild type and CNCA15 (ΔprpRBCMD) mutant strains on 3-HP and propionate. Strains were cultivated in MM supplemented with 50 mM 3-HP (a) or propionate (b) as the sole source of carbon and energy. Blue circles represent H16 wild type, red squares the CNCA15 (ΔprpRBCMD) mutant. Error bars represent the standard deviation of the mean for three independent experiments. [file 13068_2019_1489_MOESM1_ESM.docx]

**Additional file 1: Figure S1.**

**a**


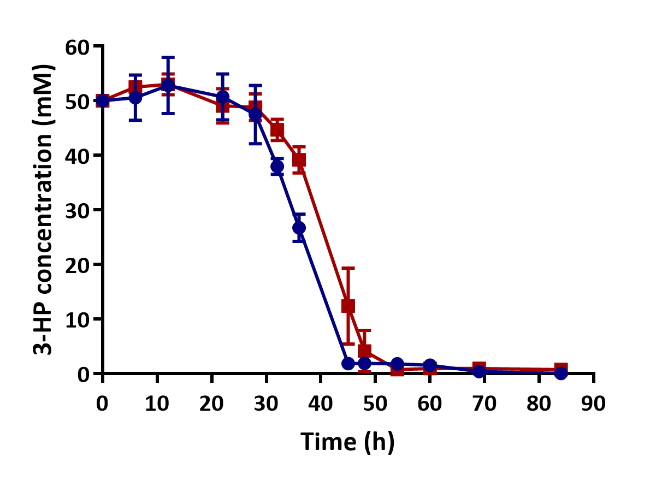

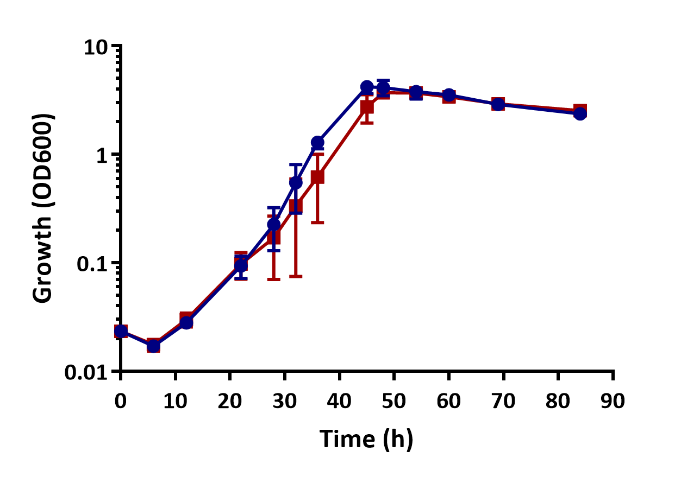


**b**


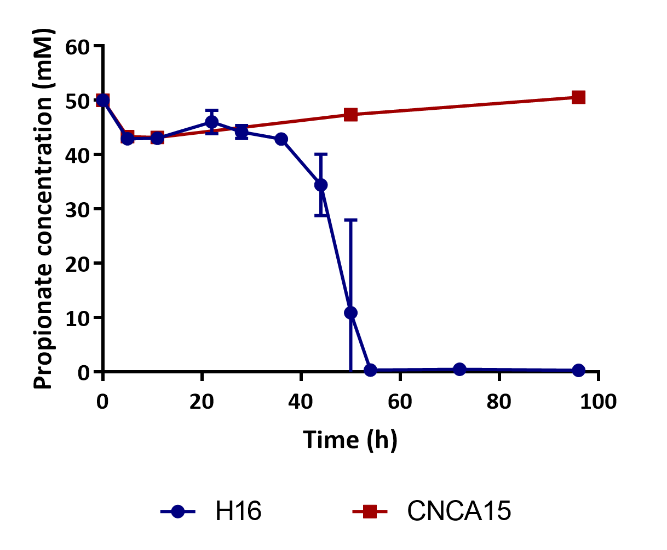

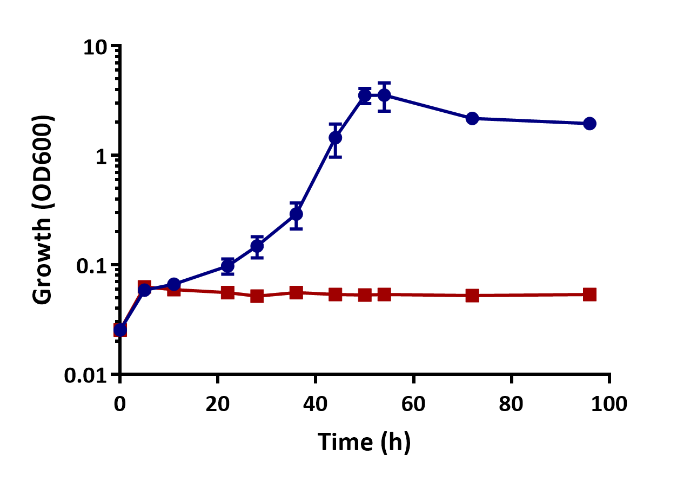


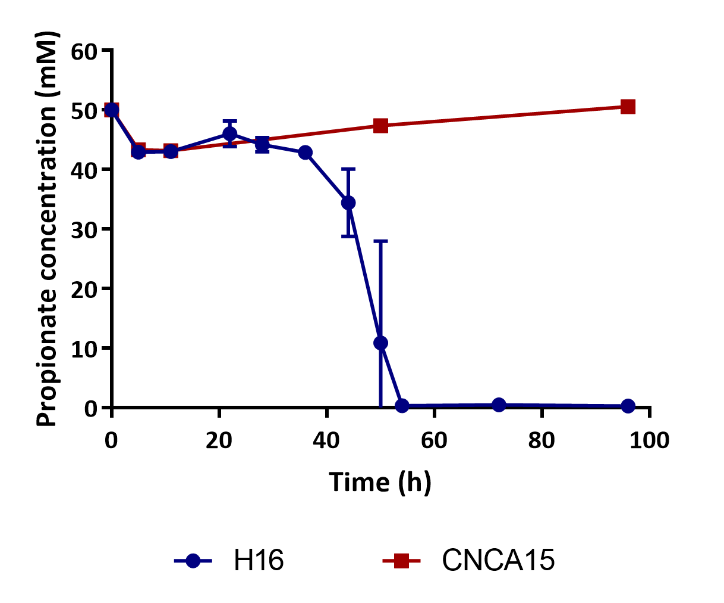


**Figure S1. Growth of *C. necator* H16 wild type and CNCA15 (Δ*prpRBCMD*) mutant strains** **on 3-HP and propionate.**

Strains were cultivated in MM supplemented with 50 mM 3-HP **(a)** or propionate **(b)** as the sole source of carbon and energy. Blue circles represent H16 wild type, red squares the CNCA15 (Δ*prpRBCMD*) mutant. Error bars represent the standard deviation of the mean for three independent experiments.
