# Supplementary material for: The genetic basis of 3-hydroxypropanoate metabolism in Cupriavidus necator H16
Source: Biotechnol Biofuels. 2019 Jun 17;12:150. doi: 10.1186/s13068-019-1489-5 (PMC6572756; doi:10.1186/s13068-019-1489-5)
Supplement: Supplementary file 2 — Additional file 2: Figure S2. Proposed valine degradation pathway in C. necator H16. (a) Valine degradation pathway with associated C. necator enzymes for each reaction. (b) Putative C. necator valine degradation operon and upstream regulatory gene, together with associated locus tags. Genes are proposed to encode the following enzymes: AraC family transcriptional regulator (araC, H16_B1193), branched-chain acyl-CoA dehydrogenase (acaD, HB161192), (methyl)malonate semialdehyde dehydrogenase (mmsA3, H16_B1191), 3-hydroxyisobutyrate dehydrogenase (hbdH, H16_B1190), enoyl-CoA dehydratase (crt, H16_B1189) and 3-hydroxyisobutyryl-CoA hydrolase (hibH; note: the gene is currently annotated to encode an enoyl-CoA hydratase/isomerase, H16_B1188). Glu, glutamate; α-KG, α-ketoglutarate. The first two steps of valine degradation are carried out by branched-chain amino acid aminotransferase and 3-methyl-2-oxobutanoate dehydrogenase (2-oxoisovalerate dehydrogenase), respectively; their encoding genes are not part of this operon. Blue colours indicate reactions and genes associated with the proposed mmsA3 operon. Sizes of genes and intergenic regions not drawn to scale. [file 13068_2019_1489_MOESM2_ESM.docx]

**Additional File 2: Figure S2.**

**a**

**
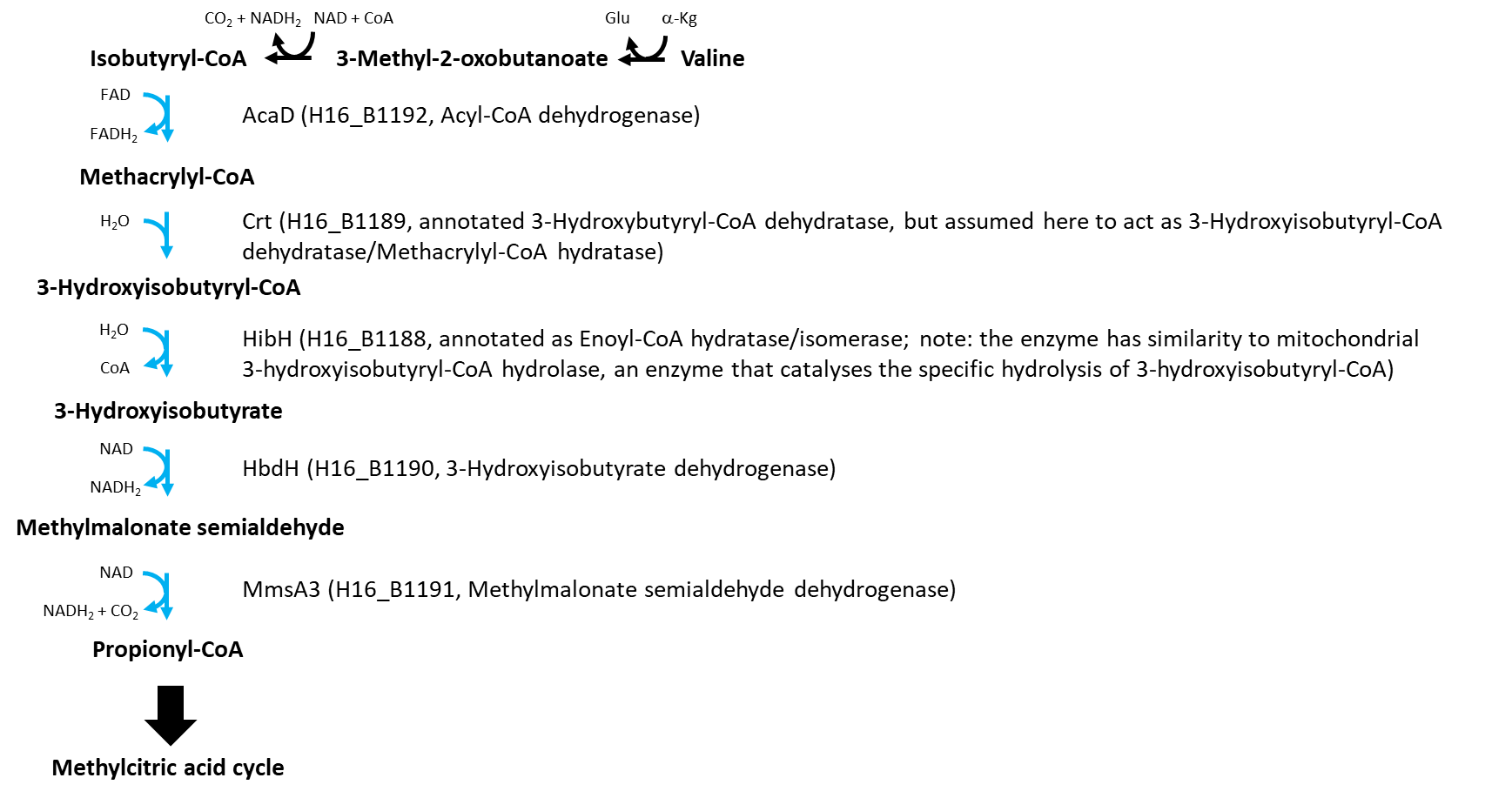
**

**b**


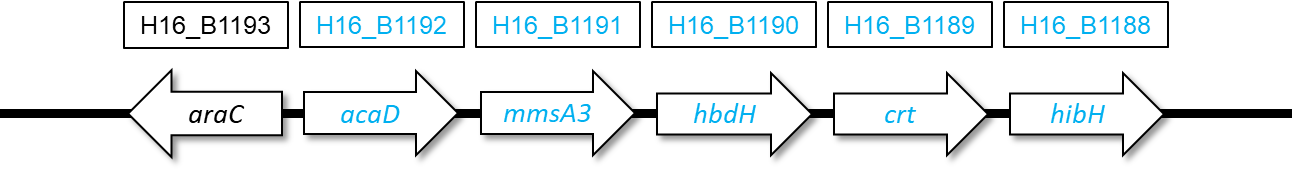


**Figure S2. Proposed valine degradation pathway in *C. necator* H16**

**(a)** Valine degradation pathway with associated *C. necator* enzymes for each reaction. **(b)** Putative *C. necator* valine degradation operon and upstream regulatory gene, together with associated locus tags. Genes are proposed to encode the following enzymes: AraC family transcriptional regulator (*araC*, H16_B1193), branched-chain acyl-CoA dehydrogenase (*acaD,* HB161192), (methy)malonate semialdehyde dehydrogenase (*mmsA3*, H16_B1191), 3-hydroxyisobutyrate dehydrogenase (*hbdH*, H16_B1190), enoyl-CoA dehydratase (*crt*, H16_B1189) and 3-hydroxyisobutyryl-CoA hydrolase (*hibH*; note: the gene is currently annotated to encode an enoyl-CoA hydratase/isomerase, H16_B1188). Glu, glutamate; α-KG, α-ketoglutarate. The first two steps of valine degradation are carried out by branched-chain amino acid aminotransferase and 3-methyl-2-oxobutanoate dehydrogenase (2-oxoisovalerate dehydrogenase), respectively; their encoding genes are not part of this operon. Blue colours indicate reactions and genes associated with the proposed *mmsA3* operon. Sizes of genes and intergenic regions not drawn to scale.
