# Supplementary material for: The genetic basis of 3-hydroxypropanoate metabolism in Cupriavidus necator H16
Source: Biotechnol Biofuels. 2019 Jun 17;12:150. doi: 10.1186/s13068-019-1489-5 (PMC6572756; doi:10.1186/s13068-019-1489-5)
Supplement: Supplementary file 3 — Additional file 3: Table S1. Genes of interest differentially expressed on 3-HP compared to fructose. [file 13068_2019_1489_MOESM3_ESM.docx]

**Table S1. Genes of interest differentially expressed on 3-HP compared to fructose**

| **Locus tag / gene** | **Encoded enzyme** | **Physiological role** | **p_adj_ value^1^** | **Fold change** |
| --- | --- | --- | --- | --- |
|  |  |  |  |  |
| **3-HP degradation** | |  |  |  |
|  | |  |  |  |
| H16_A0272 | β-Alanine-pyruvate aminotransferase | β-Alanine degradation:  *mmsA1* operon | 0.8709 | 8.2 |
| H16_A0273 | (Methyl)malonate-semialdehyde dehydrogenase MmsA1 |  | 0.2282 | 18.3 |
|  |  |  |  |  |
|  |  |  |  |  |
| H16_A3664 | (Methyl)malonate-semialdehyde dehydrogenase MmsA2 | 3-HP degradation: *mmsA2* operon | 0.0011 | 153.5 |
| H16_A3663 | 3-Hydroxypropionate dehydrogenase HpdH |  | 0.0011 | 156.4 |
|  |  |  |  |  |
| H16_B1192 | Acyl-CoA dehydrogenase | Valine degradation: *mmsA3* operon | 0.9734 | 11.3 |
| H16_B1191 | (Methyl)malonate-semialdehyde dehydrogenase MmsA3 |  | 0.8709 | 9.0 |
| H16_B1190 | 3-Hydroxyisobutyrate dehydrogenase |  | 1 | 7.5 |
| H16_B1189 | Enoyl-CoA hydratase (Methacrylyl-CoA hydratase) |  | 1 | 7.9 |
| H16_B1188 | 3-Hydroxyisobutyryl-CoA hydrolase |  | 0.8739 | 13.3 |
|  |  |  |  |  |
| **Fructose break-down and central carbon metabolism** | |  |  |  |
|  | |  |  |  |
| H16_B0278 | Fructose-bisphosphate aldolase | Gluconeogenesis | 0.9390 | 11.2 |
| H16_A2211 | Isocitrate lyase Icl1 | Glyoxylate bypass | 0.2367 | 16.8 |
| H16_A2227 | Isocitrate lyase Icl2 |  | 0.0069 | 70.9 |
| H16_B1497 | FrcR regulator | Fructose utilisation:  gene cluster for uptake and conversion to 2-keto-3-deoxy-6-phospho-D-gluconate | 0.0011 | -173.9 |
| H16_B1498 | Fructose ABC transporter FrcA |  | 0.0017 | -136.6 |
| H16_B1499 | Fructose ABC transporter FrcC |  | 0.0011 | -202.8 |
| H16_B1500 | Fructose ABC transporter FrcB |  | 0.0017 | -142.0 |
| H16_B1503 | Fructokinase |  | 0.0069 | -77.7 |
| H16_B1502 | Glucose-6-phosphate isomerase Pgi2 |  | 0.0021 | -118.4 |
| H16_B1501 | Glucose-6-phosphate 1-dehydrogenase Zwf2 |  | 0.0011 | -188.2 |
| H16_A1178 | 6-Phosphogluconate dehydratase Edd1 |  | 0.0526 | -37.8 |
| H16_A1374 | Pyruvate dehydrogenase complex, E1 component | Pyruvate dehydrogenase complex | 0.8286 | -8.7 |
| H16_A1375 | Pyruvate dehydrogenase complex, E2 component |  | 1 | -4.8 |
| H16_A1377 | Pyruvate dehydrogenase complex, E3 component |  | 1 | -6.0 |
|  |  |  |  |  |
| **Propionate metabolism** | |  |  |  |
|  | |  |  |  |
| H16_A2718 | Propionyl-CoA transferase Pct | Propionate/3-HP activation and | 1 | -3.4 |
| H16_A1904 | Propionate catabolism operon regulatory protein PrpR | Methylcitrate cylce | 1 | 1.3 |
| H16_A1905 | Methylisocitrate lyase |  | 1 | 1.4 |
| H16_A1906 | 2-Methylcitrate synthase PrpC1 |  | 1 | 1.3 |
| H16_A1907 | Fe/S-dependent 2-methylisocitrate dehydratase AcnD |  | 1 | 1.2 |
| H16_A1908 | 3-Methylitaconate isomerase |  | 1 | 2.0 |
| H16_A1909 | 2-Methylcitrate dehydratase |  | 1 | 1.9 |

Green: Gene significantly downregulated on 3-HP

Red: Gene significantly upregulated on 3-HP

Black: Change is not significant based on p_adj_ value

^1^The p_adj_ value is the p value adjusted for multiple testing according to Benjamini-Hochberg procedure
