# Supplementary material for: The genetic basis of 3-hydroxypropanoate metabolism in Cupriavidus necator H16
Source: Biotechnol Biofuels. 2019 Jun 17;12:150. doi: 10.1186/s13068-019-1489-5 (PMC6572756; doi:10.1186/s13068-019-1489-5)
Supplement: Supplementary file 4 — Additional file 4: Figure S3. Differential expression of key metabolic genes in C. necator H16 during exponential phase growth on 3-HP and fructose, respectively. Red and green boxes indicate genes significantly up- and downregulated respectively, when cells were grown on 3-HP (padj <0.05). Grey boxes indicate no statistically significant differential regulation. Locus tags are given to enable unambiguous gene identification. Abbreviations: F6P, fructose-6-phosphate; G6P, glucose-6-phosphate; 6PGL, 6-phosphogluconolactone; 6PG, 6-phosphogluconate; KDPG, 2-keto-3-deoxy-6-phosphogluconate; FBP, fructose-1,6-bisphosphate; GAP, glyceraldehyde-3-phosphate, DHAP, dihydroxyacetone phosphate; S7P, sedoheptulose-7-phosphate; R5P, ribose-5-phosphate; X5P, xylulose-5-phosphate; E4P, erythrose-4-phosphate; 1,3PG, 1,3-phosphoglycerate; 3PG, 3-phosphoglycerate; 2PG, 2-phosphoglycerate; PEP, phosphoenolpyruvate; PYR, pyruvate; AcCoA, acetyl-CoA, CIT, citrate; ISOCIT, isocitrate, AKG, α-ketoglutarate; SUCC, succinate; FUM, fumarate; MAL, malate, OAA, oxaloacetate; GLYOX, glyoxylate; 3HP, 3-hydroxypropionate; MSA, malonate semialdehyde; 3HP-CoA, 3-hydroxypropionyl-CoA; AcrCoA, acryloyl-CoA; 3Prp-CoA, propionyl-CoA; MeCIT, methylcitrate; MeISOCIT, methylisocitrate. [file 13068_2019_1489_MOESM4_ESM.docx]

**Additional File 4: Figure S3.**

**FRUCTOSE**

FRUCTOSE

F6P

G6P

6PGL

6PG

FBP

GAP

DHAP

KDPG

PYR

PEP

2PG

3PG

1,3PG

AcCoA

3HP

MSA

**3HP**

GLYOX

CIT

SUCC

OAA

MAL

FUM

2KG

SucCoA

ISOCIT

S7P

E4P

X5P

R5P

3HPCoA

AcrCoA

PrpCoA

MeCIT

OAA

MeISOCIT

PYR

H16_A1047

H16_B1502

H16_B1503

H16_B1499

H16_B1500

H16_B1498

?

H16_B1501

H16_A0316

H16_B2566

H16_B2565

H16_A1178

H16_B2567

H16_B1213

H16_A0999

H16_A1390

PHG422

H16_B0278

H16_A0568

H16_B1384

PHG416

H16_A3146

H16_B1386

PHG418

H16_A2364

H16_A2217

H16_A1188

H16_A0566

H16_B1385

PHG417

H16_A0332

H16_A0493

H16_A2038

H16_A0567

H16_A1374

H16_A1375

H16_A1377

H16_A3664

H16_B1191

H16_A0273

H16_A3663

H16_B1190

H16_A2211

H16_A2227

H16_A2627

H16_B0414

H16_B2212

H16_A2638

H16_B0568

H16_A2718

H16_A0547

H16_A0548

H16_A3056

H16_B1016

H16_B1931

H16_A2323

H16_A2324

H16_A2325

H16_A2629

H16_A2630

H16_A2631

H16_A2632

H16_A2528

H16_B0103

H16_A2634

H16_A1906

H16_A1905

H16_A3147

H16_B1388

PHG420

H16_A3711

H16_A2921

H16_A1251

H16_A1002

H16_A3153

H16_A1907

H16_A1908

H16_A1909

**Figure S3. Differential expression of key metabolic genes in *C. necator* H16 during exponential phase growth on 3-HP and fructose, respectively.** Red and green boxes indicate genes significantly up- and downregulated respectively, when cells were grown on 3-HP (padj <0.05). Grey boxes indicate no statistically significant differential regulation. Locus tags are given to enable unambiguous gene identification. Abbreviations: F6P, fructose-6-phosphate; G6P, glucose-6-phosphate; 6PGL, 6-phosphogluconolactone; 6PG, 6-phosphogluconate; KDPG, 2-keto-3-deoxy-6-phosphogluconate; FBP, fructose-1,6-bisphosphate; GAP, glyceraldehyde-3-phosphate, DHAP, dihydroxyacetone phosphate; S7P, sedoheptulose-7-phosphate; R5P, ribose-5-phosphate; X5P, xylulose-5-phosphate; E4P, erythrose-4-phosphate; 1,3PG, 1,3-phosphoglycerate; 3PG, 3-phosphoglycerate; 2PG, 2-phosphoglycerate; PEP, phosphoenolpyruvate; PYR, pyruvate; AcCoA, acetyl-CoA, CIT, citrate; ISOCIT, isocitrate, AKG, α-ketoglutarate; SUCC, succinate; FUM, fumarate; MAL, malate, OAA, oxaloacetate; GLYOX, glyoxylate; 3HP, 3-hydoxypropionate; MSA, malonate semialdehyde; 3HP-CoA, 3-hydoxypropionyl-CoA; AcrCoA, acryloyl-CoA; 3Prp-CoA, propionyl-CoA; MeCIT, methylcitrate; MeISOCIT, methylisocitrate.
