# Supplementary material for: The genetic basis of 3-hydroxypropanoate metabolism in Cupriavidus necator H16
Source: Biotechnol Biofuels. 2019 Jun 17;12:150. doi: 10.1186/s13068-019-1489-5 (PMC6572756; doi:10.1186/s13068-019-1489-5)
Supplement: Supplementary file 5 — Additional file 5: Figure S4. Growth (a) and 3-HP consumption (b) of C. necator strains CNCA07 (ΔhpdH) and CNCA16 (ΔhbdH). Strains H16, CNCA07 (ΔhpdH) and CNCA16 (ΔhbdH) were cultivated in MM supplemented with 50 mM 3-HP as the sole source of carbon and energy. Blue circles, H16 wild type; inverted purple triangles, CNCA07; pink diamonds, CNCA16. Error bars represent the standard deviation of the mean for three independent experiments. [file 13068_2019_1489_MOESM5_ESM.docx]

**Additional file 5: Figure S4**

**b**

**a**


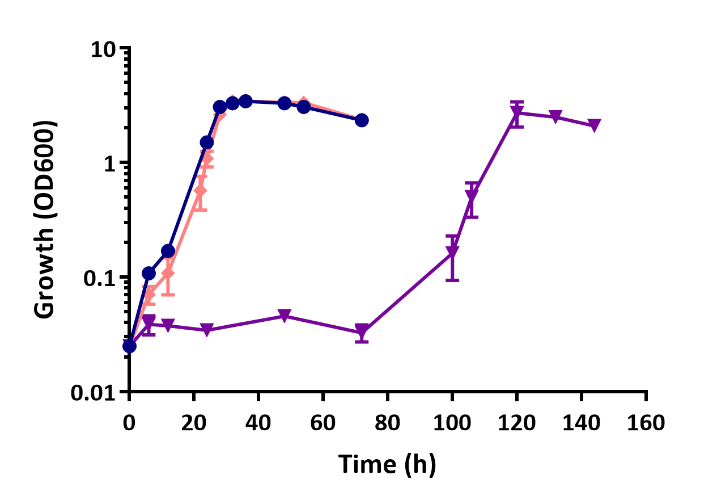

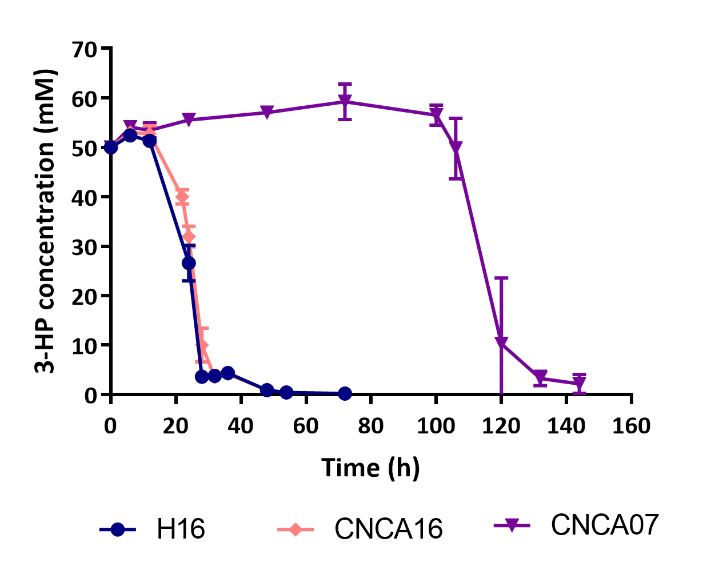

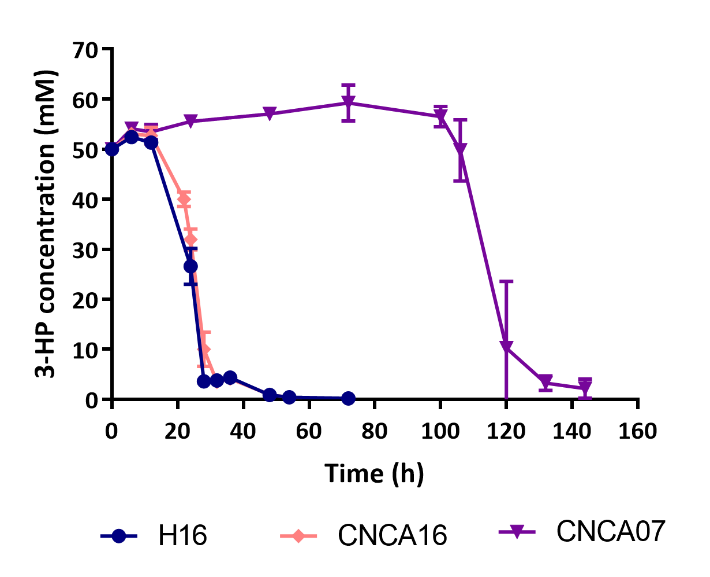


**Figure S4. Growth (a) and 3-HP consumption (b) of *C. necator* strains CNCA07 (Δ*hpdH*) and CNCA16 (Δ*hbdH*)**

Strains H16, CNCA07 (Δ*hpdH*) and CNCA16 (Δ*hbdH*) were cultivated in MM supplemented with 50 mM 3-HP as the sole source of carbon and energy. Blue circles, H16 wild type; inverted purple triangles, CNCA07; pink diamonds, CNCA16. Error bars represent the standard deviation of the mean for three independent experiments.
