# Supplementary material for: The genetic basis of 3-hydroxypropanoate metabolism in Cupriavidus necator H16
Source: Biotechnol Biofuels. 2019 Jun 17;12:150. doi: 10.1186/s13068-019-1489-5 (PMC6572756; doi:10.1186/s13068-019-1489-5)
Supplement: Supplementary file 6 — Additional file 6: Figure S5. Cell growth (a) and 3-HP consumption (b) of C. necator CNCA13 complementation strains. The different CNCA13 (ΔmmsA1ΔmmsA2ΔmmsA3) complementation strains carrying plasmids pBBR1MCS-2-PphaC-mmsA1 (green triangles), pBBR1MCS-2-PphaC-mmsA2 (inverted purple triangles), and pBBR1MCS-2-PphaC-mmsA3 (orange diamonds), respectively, were cultivated in MM supplemented with 50 mM 3-HP as the sole source of carbon and energy. Controls included H16 wild type (blue circles) and CNCA13 (brown squares) carrying the empty pBBR1MCS-2-PphaC vector. Error bars indicate the standard deviation of the mean for three independent experiments. [file 13068_2019_1489_MOESM6_ESM.docx]

**Additional file 6: Figure S5.**

**a**


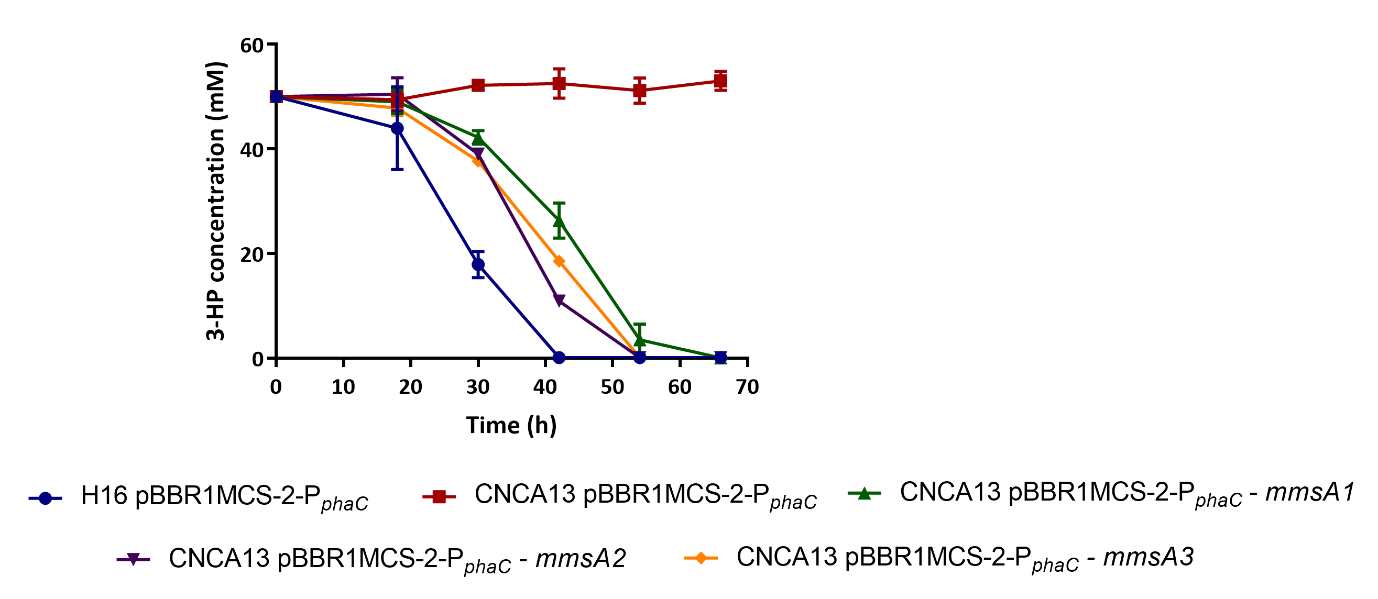

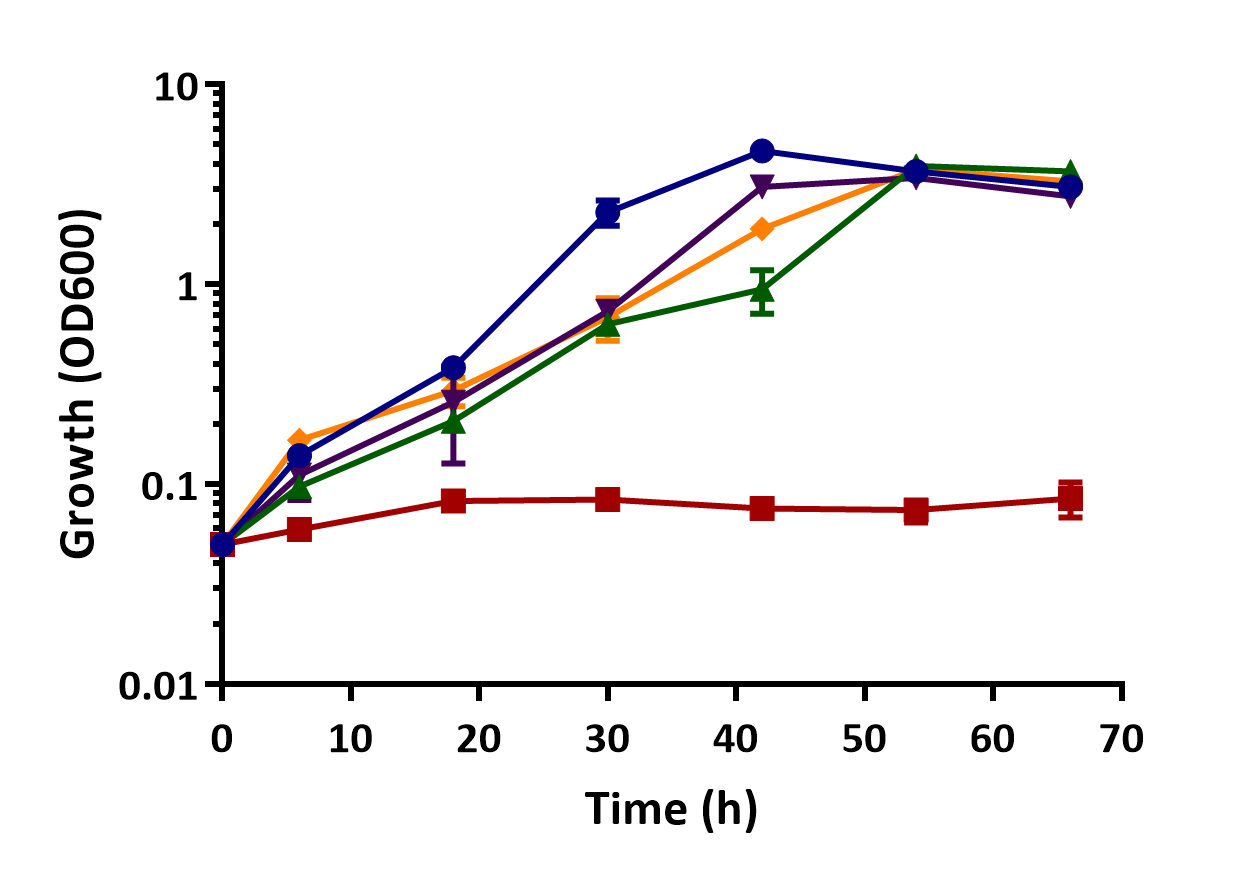


**b**


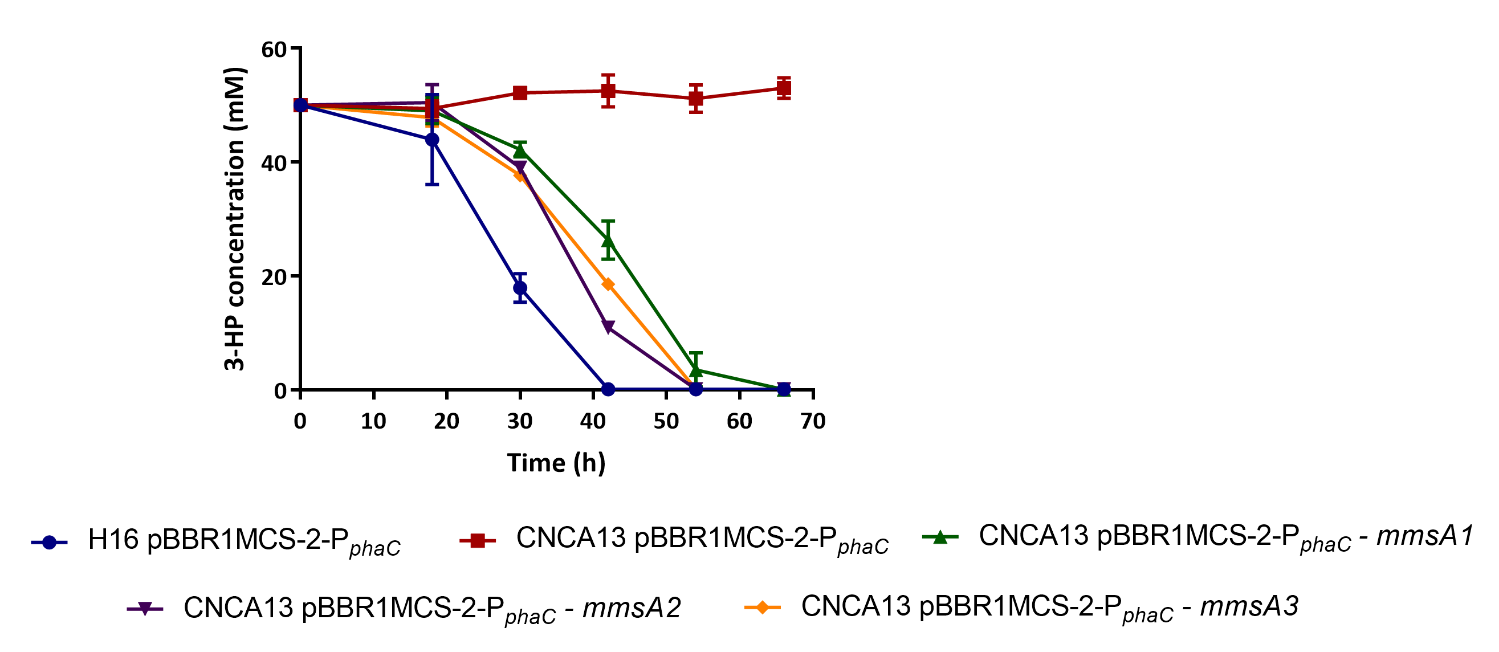


**Figure S5. Cell growth (a) and 3-HP consumption (b) of *C. necator* CNCA13 complementation strains**

The different CNCA13 (Δ*mmsA1*Δ*mmsA2*Δ*mmsA3*) complementation strains carrying plasmids pBBR1MCS-2-P*_phaC_*-*mmsA1* (green triangles), pBBR1MCS-2-P*_phaC_*-*mmsA2* (inverted purple triangles), and pBBR1MCS-2-P*_phaC_*-*mmsA3* (orange diamonds), respectively, were cultivated in MM supplemented with 50 mM 3-HP as the sole source of carbon and energy. Controls included H16 wild type (blue circles) and CNCA13 (brown squares) carrying the empty pBBR1MCS-2-P*_phaC_* vector. Error bars indicate the standard deviation of the mean for three independent experiments.
