# Supplementary material for: The genetic basis of 3-hydroxypropanoate metabolism in Cupriavidus necator H16
Source: Biotechnol Biofuels. 2019 Jun 17;12:150. doi: 10.1186/s13068-019-1489-5 (PMC6572756; doi:10.1186/s13068-019-1489-5)
Supplement: Supplementary file 7 — Additional file 7: Figure S6. Genetic complementation of CNCA16 (Δhbdh), CNCA05 (ΔmmsA3) and CNCA13 (ΔmmsA1ΔmmsA2ΔmmsA3) mutant strains restores growth on valine. (a) Growth of C. necator H16 wild type, CNCA16 (ΔhbdH) mutant and complemented CNCA16 (ΔhbdH) mutant. Strain H16 (A) and the CNCA16 mutant control (B) contained empty pMTL71301 vector, the complemented CNCA16 mutant (C) contained plasmid pMTL71301-PacaD-hbdh. (b) Growth of C. necator H16 wild type, CNCA05 (ΔmmsA3) mutant and complemented CNCA05 (ΔmmsA3) mutant. Strain H16 (A) and the CNCA05 mutant control (B) contained empty pBBR1MCS-2-PphaC-eyfp vector, the complemented CNCA05 mutant (C) contained plasmid pBBR1MCS-2-PphaC-mmsA3. (c) Growth of C. necator H16 CNCA13 (ΔmmsA1ΔmmsA2ΔmmsA3) complemented with either mmsA1, mmsA2 and mmsA3 genes. Strain H16 (A) and the CNCA13 mutant control (B) contained empty pBBR1MCS-2-PphaC-1-eyfp vector, whereas the complemented CNCA13 mutants contained pBBR1MCS-2-PphaC-mmsA1 (C), pBBR1MCS-2-PphaC-mmsA2 (D) and pBBR1MCS-2-PphaC-mmsA3 (E), respectively. All strains were grown on MM agar plates containing 25 mM fructose (i) and 30 mM valine (ii). Agar plates were incubated at 30 °C for 5 days. [file 13068_2019_1489_MOESM7_ESM.docx]

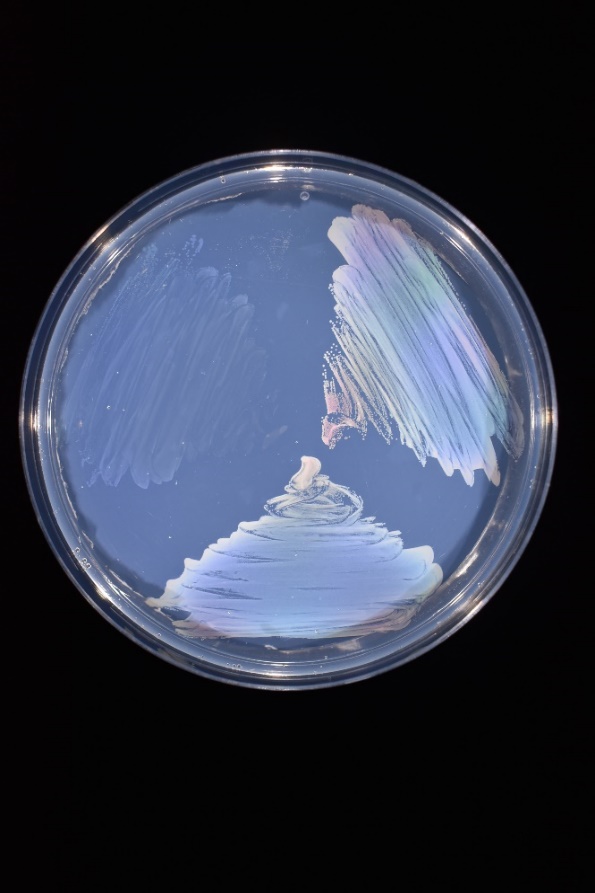
**Additional file 7: Figure S6.**

**a**

**i**


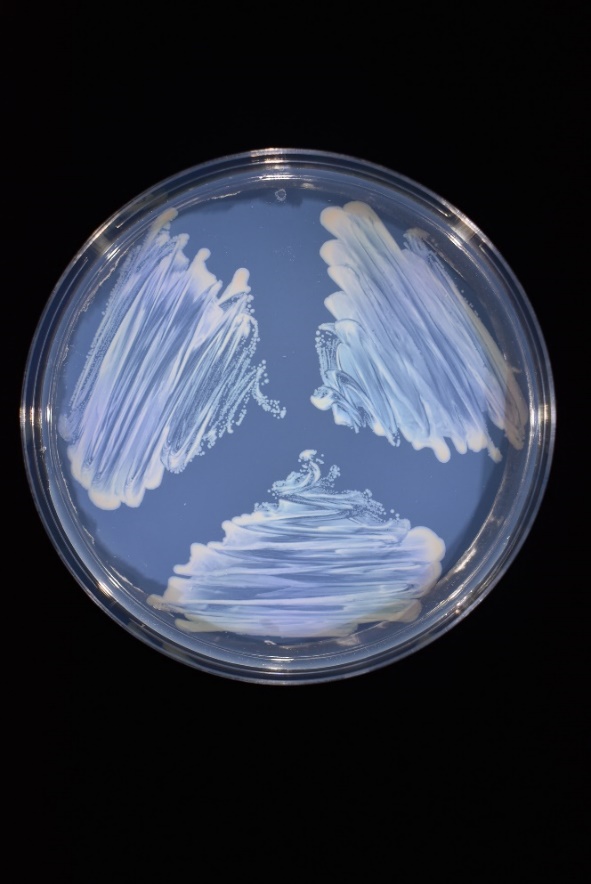


**C**

**B**

**C**

**B**

**ii**

**A**

**A**


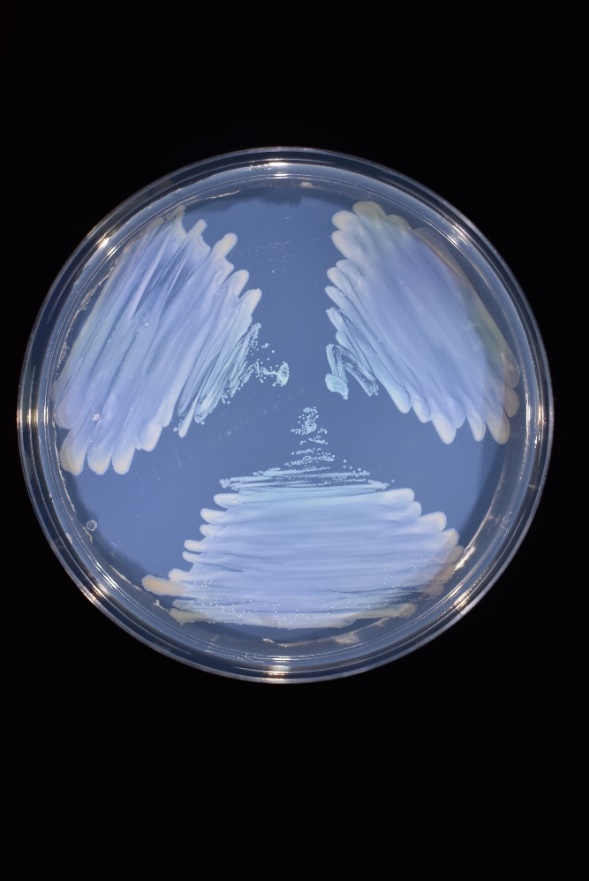

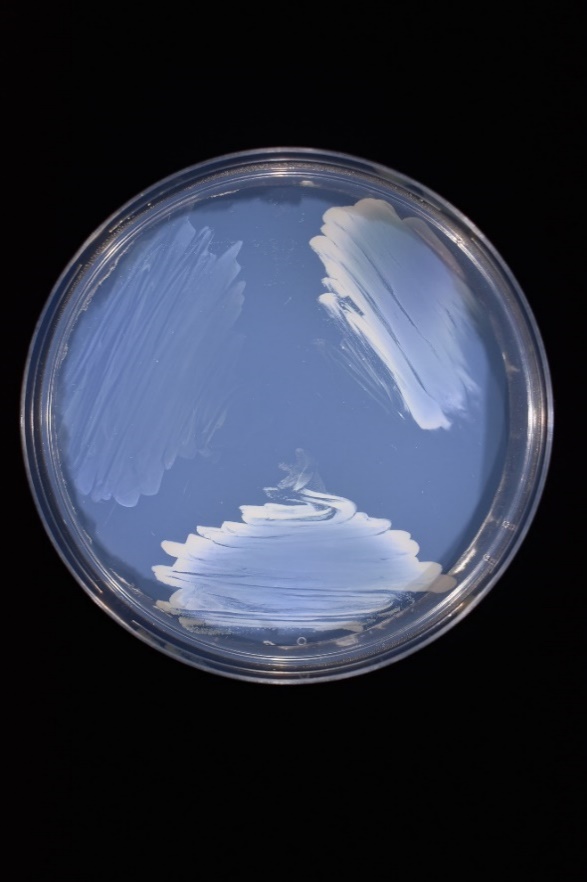


**b**

**i**

**B**

**C**

**B**

**A**

**ii**

**C**

**A**


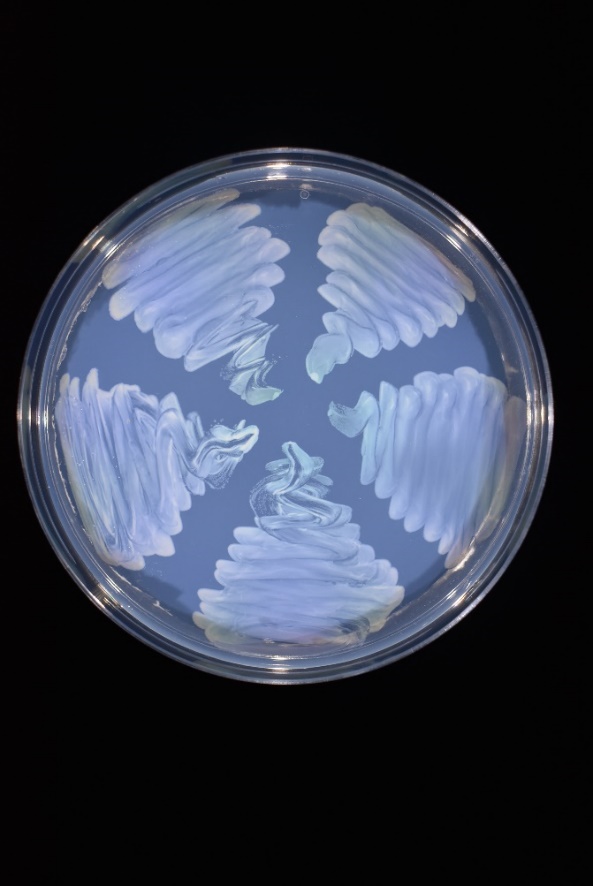

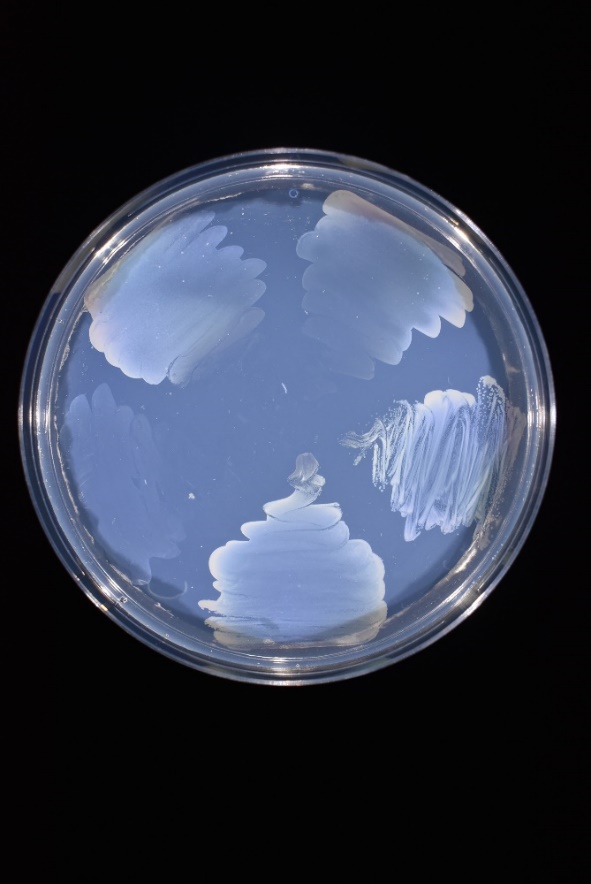


**E**

**D**

**A**

**E**

**B**

**B**

**C**

**C**

**D**

**i**

**c**

**ii**

**A**

**Figure S6. Genetic complementation of CNCA16 (*Δhbdh*), CNCA05 (Δ*mmsA*3) and CNCA13 (*ΔmmsA1ΔmmsA2ΔmmsA3*) mutant strains restores growth on valine**

**(a)** Growth of *C. necator* H16 wild type, CNCA16 (Δ*hbdH*) mutant and complemented CNCA16 (Δ*hbdH*) mutant. Strain H16 (A) and the CNCA16 mutant control (B) contained empty pMTL71301 vector, the complemented CNCA16 mutant (C) contained plasmid pMTL71301-P*_acaD_*-*hbdh.* **(b)** Growth of *C. necator* H16 wild type, CNCA05 (Δ*mmsA*3) mutant and complemented CNCA05 (Δ*mmsA*3) mutant. Strain H16 (A) and the CNCA05 mutant control (B) contained empty pBBR1MCS-2-P*_phaC_*-*eyfp* vector, the complemented CNCA05 mutant (C) contained plasmid pBBR1MCS-2-P*_phaC_*-*mmsA3.* **(c)** Growth of *C. necator* H16 CNCA13 (Δ*mmsA1*Δ*mmsA2*Δ*mmsA3*) complemented with either *mmsA1*, *mmsA2* and *mmsA3* genes. Strain H16 (A) and the CNCA13 mutant control (B) contained empty pBBR1MCS-2-P*_phaC_*-*1*-*eyfp* vector, whereas the complemented CNCA13 mutants contained pBBR1MCS-2-P*_phaC_*-*mmsA1* (C), pBBR1MCS-2-P*_phaC_*-*mmsA2* (D) and pBBR1MCS-2-P*_phaC_*-*mmsA3* (E), respectively. All strains were grown on MM agar plates containing 25 mM fructose (i) and 30 mM valine (ii). Agar plates were incubated at 30°C for 5 days.
