# Supplementary material for: The genetic basis of 3-hydroxypropanoate metabolism in Cupriavidus necator H16
Source: Biotechnol Biofuels. 2019 Jun 17;12:150. doi: 10.1186/s13068-019-1489-5 (PMC6572756; doi:10.1186/s13068-019-1489-5)
Supplement: Supplementary file 8 — Additional file 8: Figure S7. Growth of C. necator H16 and CNCA15 (ΔprpRBCMD) mutant strains on fructose and valine. Strains H16 (A) and CNCA15 (B) were grown on MM agar plates containing 25 mM fructose (a, left panel) and 30 mM valine (b, right panel). Agar plates were incubated at 30 °C for 5 days. [file 13068_2019_1489_MOESM8_ESM.docx]

**Additional file 8: Figure S7.**


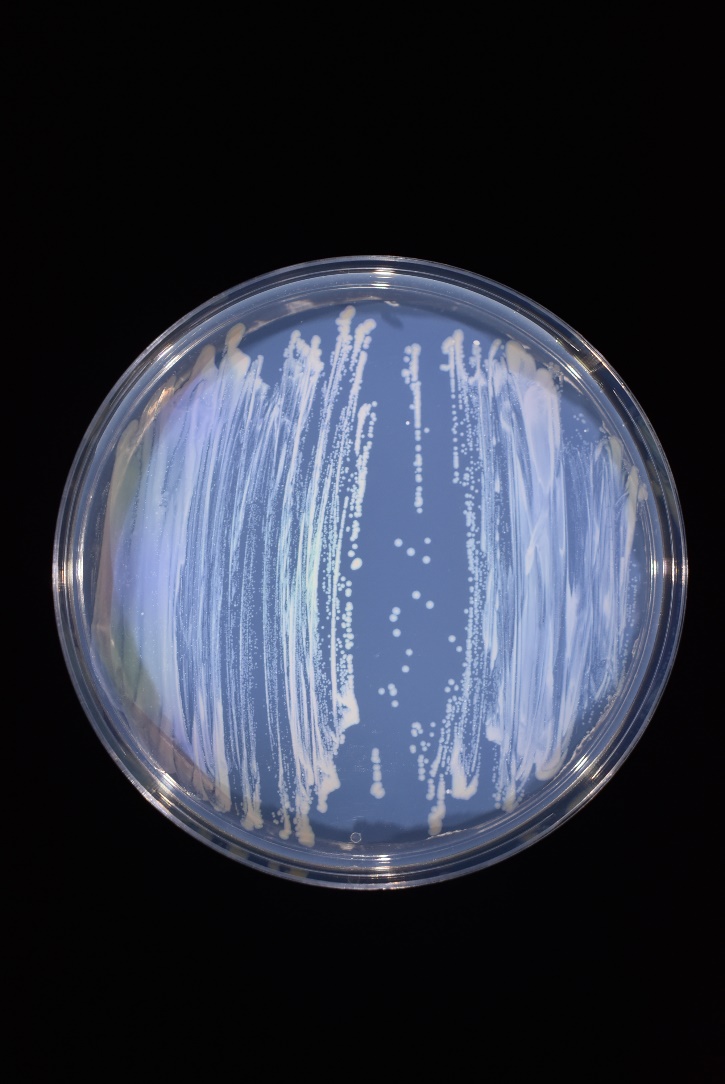

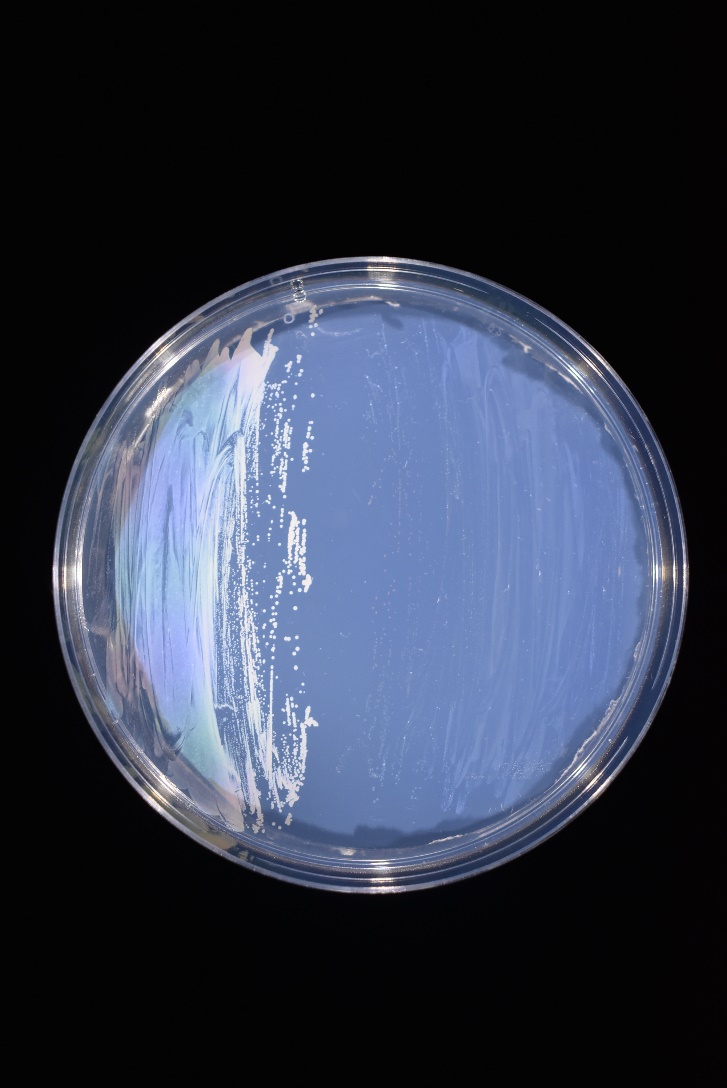


**B**

**B**

**A**

**A**

**b**

**a**

**Figure S7. Growth of *C. necator* H16 and CNCA15 (Δ*prpRBCMD*) mutant strains on fructose and valine**. Strains H16 (A) and CNCA15 (B) were grown on MM agar plates containing 25 mM fructose **(a, left panel)** and 30 mM valine **(b, right panel)**. Agar plates were incubated at 30°C for 5 days.
