# Supplementary material for: The genetic basis of 3-hydroxypropanoate metabolism in Cupriavidus necator H16
Source: Biotechnol Biofuels. 2019 Jun 17;12:150. doi: 10.1186/s13068-019-1489-5 (PMC6572756; doi:10.1186/s13068-019-1489-5)
Supplement: Supplementary file 9 — Additional file 9: Table S2. Strains used and generated in this study. [file 13068_2019_1489_MOESM9_ESM.docx]

**Table S2. Strains used and generated in this study**

| Strain | Genotype / Description^a^ | Reference or source |
| --- | --- | --- |
|  |  |  |
| ***E. coli*** |  |  |
| DH5α | F^-^ φ80*lac*ZΔM15 Δ(*lac*ZYA-*arg*F)U169 *deo*R *recA1* *endA1 hsdR17*(r_k_^-^, m_k_^+^) *pho*A *sup*E44 *thi*-1 *gyr*A96 *relA1* λ- | (1) |
| S17-1 λpir | *recA thi pro* *hsdR^-^ hsdM^+^* RP4::2-Tc::Mu::Km Tn7 *λpir*, Tp^R^ Sm^R^ | (2) |
| ***C. necator*** |  |  |
| H16 | H16 wild type strain (DSM 428) | Leibniz Institute DSMZ-German Collection of Micro-organisms and Cell Cultures, Braunschweig, Germany |
| CNCA03 | H16 Δ*mmsA1* (entire ORF) deletion mutant | This study |
| CNCA04 | H16 Δ*mmsA2* (entire ORF) deletion mutant | This study |
| CNCA05 | H16 Δ*mmsA3* (entire ORF) deletion mutant | This study |
| CNCA06 | H16 Δ*mcd* (entire ORF) deletion mutant | This study |
| CNCA07 | H16 Δ*hpdH* (entire ORF) deletion mutant | This study |
| CNCA08 | H16 Δ*mmsA2*Δ*mmsA3* (entire ORFs) deletion mutant, constructed from CNCA04 | This study |
| CNCA09 | H16 Δ*mmsA2*Δ*mcd* (entire ORFs) deletion mutant, constructed from CNCA04 | This study |
| CNCA10 | H16 Δ*mmsA2*Δ*hpdH* (entire ORF) deletion mutant | This study |
| CNCA11 | H16 Δ*mmsA3*Δ*hbdh* (entire ORF) deletion mutant | This study |
| CNCA12 | H16 Δ*mmsA1*Δ*mmsA2* (entire ORFs) deletion mutant, constructed from CNCA04 | This study |
| CNCA13 | H16 Δ*mmsA1*Δ*mmsA2*Δ*mmsA3* (entire ORFs) deletion mutant, constructed from CNCA12 | This study |
| CNCA15 | H16 Δ*prpRBCMD* (entire gene cluster) deletion mutant | This study |
| CNCA16 | H16 Δ*hbdH* (entire ORF) deletion mutant | This study |

1. Grant SG, Jessee J, Bloom FR, Hanahan D. Differential plasmid rescue from transgenic mouse DNAs into Escherichia coli methylation-restriction mutants. Proc Natl Acad Sci USA. 1990;87(12):4645-9.

2. Simon R, Priefer U, Pühler A. A broad host range mobilization system for invivo genetic engineering: transposon mutagenesis in Gram-negative bacteria. Bio/Technology. 1983;1:784-91.
