# Supplementary material for: The genetic basis of 3-hydroxypropanoate metabolism in Cupriavidus necator H16
Source: Biotechnol Biofuels. 2019 Jun 17;12:150. doi: 10.1186/s13068-019-1489-5 (PMC6572756; doi:10.1186/s13068-019-1489-5)
Supplement: Supplementary file 10 — Additional file 10: Table S3. Plasmids used and generated in this study. [file 13068_2019_1489_MOESM10_ESM.docx]

**Table S3. Plasmids used and generated in this study**

| Plasmid | Genotype / Description^*^ | Reference or source |
| --- | --- | --- |
| pLO3 | Suicide vector used for deletion of genes in *C. necator*; carries *sacB* gene and Tet resistance cassette | (1); obtained from  Dieter Jendrossek, University of Stuttgart |
| pLO3-Δ*mmsA1* | pLO3 derivative for deletion of *mmsA1* | This study |
| pLO3-Δ*mmsA2* | pLO3 derivative for deletion of *mmsA2* | This study |
| pLO3-Δ*mmsA3* | pLO3 derivative for deletion of *mmsA3* | This study |
| pLO3-Δ*mcd* | pLO3 derivative for deletion of *mcd* | This study |
| pLO3-Δ*hpdH* | pLO3 derivative for deletion of *hpdH* | This study |
| pLO3-Δ*hbdH* | pLO3 derivative for deletion of *hbdH* | This study |
| pLO3-Δ*mmsA2*Δ*hpdH* | pLO3 derivative for combined deletion of *mmsA2* and *hpdH* | This study |
| pLO3-Δ*mmsA3*Δ*hbdH* | pLO3 derivative for combined deletion of *mmsA2* and *hbdH* | This study |
| pLO3-Δ*prpRBCMD* | pLO3 derivative for deletion of the *prp* operon including *prpR*, *prpB*, *prpC*, *acnM*, ORF5 and *prpD* | This study |
| pBBR1MCS-2-P*_phaC_*-*eyfp-c1* | Broad host range, medium-copy-number plasmid for construction of fusions C-terminal to eYfp under the control of the *phaCAB* promoter, confers Km resistance; used here to obtain pBBR1MCS-2-P_phaC_ derivatives following removal of *eyfp-c1* | (2); obtained from  Dieter Jendrossek, University of Stuttgart |
| pBBR1MCS-2-P*_phaC_*-*mmsA1* | pBBR1MCS-2-P_phaC_ derivative with *C. necator* H16 *mmsA1* under control of the *phaCAB* promoter | This study |
| pBBR1MCS-2-P*_phaC_*- *mmsA2* | pBBR1MCS-2-P_phaC_ derivative with *C. necator* H16 *mmsA2* under control of the *phaCAB* promoter | This study |
| pBBR1MCS-2-P*_phaC_*- *mmsA3* | pBBR1MCS-2-P_phaC_ derivative with *C. necator* H16 *mmsA3* under control of the *phaCAB* promoter | This study |
| pMTL71301 | Broad host range modular vector for gene expression in *C. necator*, pBBR1 origen of replication, confers Tet resistance | Muhammad Ehsaan, University of Nottingham, unpublished |
| pMTL71301-P*_acaD_*- *hbdh* | pMTL71301 derivative with *C. necator* H16 *hbdh* under control of the native P*_acaD_* promoter (upstream of the *mmsA3* operon) | This study |

1. Lenz O, Friedrich B. A novel multicomponent regulatory system mediates H_2_ sensing in *Alcaligenes eutrophu*s. Proc Natl Acad Sci USA. 1998;95(21):12474-9.

2. Pfeiffer D, Jendrossek D. Interaction between poly(3-hydroxybutyrate) granule-associated proteins as revealed by two-hybrid analysis and identification of a new phasin in *Ralstonia eutropha* H16. Microbiology. 2011;157(Pt 10):2795-807.
