# Supplementary material for: The genetic basis of 3-hydroxypropanoate metabolism in Cupriavidus necator H16
Source: Biotechnol Biofuels. 2019 Jun 17;12:150. doi: 10.1186/s13068-019-1489-5 (PMC6572756; doi:10.1186/s13068-019-1489-5)
Supplement: Supplementary file 11 — Additional file 11: Table S4. Primers used in this study. [file 13068_2019_1489_MOESM11_ESM.docx]

**Table S4. Primers used in this study**

| ^Primer^ | ^Sequence (5’ to 3’)^ | ^Description^ |
| --- | --- | --- |
|  |  |  |
| pLO3_ KO_*mmsA2hpdH*_GA_LHA_Fw | AGATCCTTTTAATTCGAGCTCAGCAAGTCGTCTTGCCCGC | Primers used for amplification of the *mmsA2hpdH* upstream region |
| pLO3_KO_*mmsA2hpdH*_GA_LHA_Rv | AGCAGGGACACATGAGCGCGCCACGGGT |  |
| pLO_ KO_*mmsA2hpdH*_GA_RHA_Fw | CGCGCTCATGTGTCCCTGCTGCTGCAGG | Primers used for amplification of the *mmsA2hpdH* downstream region |
| pLO3_KO_*mmsA2hpdH*_GA_RHA_Rv | GCAGGTTTAAACAGTCGACTCTAGAGCGGGACAGAGTGCAAGG |  |
| pLO3_*mmsA2hpdH*_KO_HA_seq_out_Fw | CTCCAGGAACTTGGTGAGCAGGTTG | Primers used for sequencing outside the *mmsA2hpdH* coding region |
| pLO3_*mmsA2hpdH*_KO_HA_seq_out_Rv | CTTGAGCTGCGCATCGCTGAACTGG |  |
| pLO3_KO_*mmsA3hbhH*_GA_LHA_Fw | AGATCCTTTTAATTCGAGCTCGTGGCGGCATCCACGCGC | Primers used for amplification of the *mmsA3hbhH* upstream region |
| pLO3_KO_*mmsA3hbhH*_GA_LHA_Rv | GGAGACAACCATGATCGAGTTCGCCCTGCACG |  |
| pLO3_KO_*mmsA3hbhH*_GA_RHA_Fw | ACTCGATCATGGTTGTCTCCGGAAGGTC | Primers used for amplification of the *mmsA3hbhH* downstream region |
| pLO3_KO_*mmsA3hbhH*_GA_RHA_Rv | GCAGGTTTAAACAGTCGACTCTAGAATGCACAGCGACTACACC |  |
| pLO3_*mmsA3hbhH*_KO_HA_seq_out_Fw | GCATCGTCGAACAGGTCGACAAAGC | Primers used for sequencing outside the *mmsA3hbhH* coding region |
| pLO3_*mmsA3hbhH*_KO_HA_seq_out_Rv | CGAAGATTGGCGGATTTTGGCATAGG |  |
| pLO3_KO_*mmsA3*_GA_LHA_Fw | AGATCCTTTTAATTCGAGCTCGCCCCATGCCGGCCAGCA | Primers used for amplification of the *mmsA3* upstream region |
| pLO3_KO_*mmsA3*_GA_LHA_Rv | GGAGACAACCTCGGGCGGCATTCAAGAACGACAC |  |
| pLO3_KO_*mmsA3*_GA_RHA_Fw | TGCCGCCCGAGGTTGTCTCCGGAAGGTC | Primers used for amplification of the *mmsA3* downstream region |
| pLO3_KO_*mmsA3*_GA_RHA_Rv | GCAGGTTTAAACAGTCGACTCTAGAATGCACAGCGACTACACC |  |
| pLO3_*mmsA3*_KO_HA_seq_out_Fw | CGAGGATCAGGTTGTTGCAG | Primers used for sequencing outside *mmsA3* coding region |
| pLO3_*mmsA3*_KO_HA_seq_out_Rv | GGCGGATTTTGGCATAGGAG |  |
| pLO3_*mmsA3*_KO_HA_seq_in_Fw | GTATCGACGCCGATCACCACCAGC | Primers used for sequencing inside the *mmsA3* coding region |
| pLO3_*mmsA3*_KO_HA_seq_in_Rv | CGAATTCCGCAACGTGGTCAATCC |  |
| pLO3_KO_*mmsA1*_GA_LHA_Fw | AGATCCTTTTAATTCGAGCTCCGATCGAGCTGCTGCATG | Primers used for amplification of the *mmsA1* upstream region |
| pLO3_KO_*mmsA1*_GA_LHA_Rv | CGCTCTCCCCTTGGGATTTCCTTCGATAGC |  |
| pLO3_KO_*mmsA1*_GA_RHA_Fw | GAAATCCCAAGGGGAGAGCGGGCATGAA | Primers used for amplification of the *mmsA1* downstream region |
| pLO3_KO_*mmsA1*_GA_RHA_Rv | GCAGGTTTAAACAGTCGACTCTAGACTGGTGCTTCCCAACGTC |  |
| pLO3_*mmsA1*_KO_HA_seq_out_Fw | CTACCTGGAAAAGCTGCGTG | Primers used for sequencing outside the *mmsA1* coding region |
| pLO3_*mmsA1*_KO_HA_seq_out_Rv | CGCGATGACTACCGCTTATC |  |
| pLO3_*mmsA1*_KO_HA_seq_in_Fw | CATCGAGGTGGTGGAATTCGCTTG | Primers used for sequencing inside the *mmsA1* coding region |
| pLO3_*mmsA1*_KO_HA_seq_in_Rv | GCTCTTGTAGCGCGTGTAGAAACGC |  |
| pLO3_KO_*mcd*_GA_LHA_Fw | AGATCCTTTTAATTCGAGCTCTTGTAGAACGACGGCGCAATCG | Primers used for amplification of the *mcd* upstream region |
| pLO3_KO_*mcd*_GA_LHA_Rv | CAGGTACCGCCCCTGGTCCGGTCACGCG |  |
| pLO3_KO_*mcd*_GA_RHA_Fw | CGGACCAGGGGCGGTACCTGCCTTGATGCATCG | Primers used for amplification of the *mcd* downstream region |
| pLO3_KO_*mcd*_GA_RHA_Rv | GCAGGTTTAAACAGTCGACTCTAGAGGGCCAGCGCCGGCAATC |  |
| pLO3_*mcd*_KO_HA_seq_out_Fw | AGTTCAGCTTGCCAGGATTG | Primers used for sequencing outside the *mcd* coding region |
| pLO3_*mcd*_KO_HA_seq_out_Rv | CACTTCACGTAAAGCCAGGG |  |
| pLO3_*mcd*_KO_HA_seq_in_Fw | CAAGCGTGCCAGATTGTCATCCAG | Primers used for sequencing inside of the *mcd* region |
| pLO3_*mcd*_KO_HA_seq_in_Rv | GTGCATGAAATCGCCTCATGGACC |  |
| pLO3_KO_*mmsA2*_GA_LHA_Fw | AGATCCTTTTAATTCGAGCTCCGCTCCAGGATGTCCCAGCG | Primers used for amplification of the *mmsA2* upstream region |
| pLO3_KO_*mmsA2*_GA_LHA_Rv | AGCCAGCGCGGCCGCAACGATGACGACAAG |  |
| pLO3_KO_*mmsA2*_GA_RHA_Fw | TCGTTGCGGCCGCGCTGGCTTCTGCAAGGA | Primers used for amplification of the mmsA2 downstream region |
| pLO3_KO_*mmsA2*_GA_RHA_Rv | GCAGGTTTAAACAGTCGACTCTAGACTAAGTCCGGAACACCGACTCG |  |
| pLO3_*mmsA2*_KO_HA_seq_out_Fw | CTGGTTCACTTCGAAATAGCCGACG | Primers used for sequencing outside the *mmsA2* coding region |
| pLO3_*mmsA2*_KO_HA_seq_out_Rv | GCTACCACGAGATCTTCAATGAAGC |  |
| pLO3_*mmsA2*_KO_HA_seq_in_Fw | GATCGGCACATTGATGCCGACCATG | Primers used for sequencing inside the *mmsA2* coding region |
| pLO3_*mmsA2*_KO_HA_seq_in_Rv | GCATCCTGTTCAAGTTCAAGGAGCTG |  |
| pLO3_KO_*hpdH*_GA_LHA_Fw | AGATCCTTTTAATTCGAGCTCAGCGATCGACGAGACCACGCTCATGTTG | Primers used for amplification of the *hpdH* upstream region |
| pLO3_KO_*hpdH*_GA_LHA_Rv | AAGAACAGCGCATGAGCGCGCCACGGGTCGCG |  |
| pLO3_KO_*hpdH*_GA_RHA_Fw | CGCGCTCATGCGCTGTTCTTGTCGTCATCGTTGC | Primers used for amplification of the *hpdH* downstream region |
| pLO3_KO_*hpdH*_GA_RHA_Rv | GCAGGTTTAAACAGTCGACTCTAGAGTCGGAACGTGATCCGTCGCCG |  |
| pLO3_*hpdH*_KO_HA_seq_out_Fw | ACGCTGCAGCACGGTGATCTTGTC | Primers used for sequencing outside the *hpdH* coding region |
| pLO3_*hpdH*_KO_HA_seq_out_Rv | pLO3_mmsA2_KO_HA_seq_in_Rv |  |
| pLO3_*hpdH*_KO_HA_seq_in_Fw | pLO3_mmsA2_KO_HA_seq_out_Fw | Primers used for sequencing inside the *hpdH* coding region |
| pLO3_*hpdH*_KO_HA_seq_in_Rv | GGAGACATTCGACTACATCATCGTGG |  |
| pLO3_KO_*hbdH*_GA_LHA_Fw | AGATCCTTTTAATTCGAGCTCCCTTGGGGCCGGCGCCGCGCAGCAC | Primers used for amplification of the *hbdH* upstream region |
| pLO3_KO_*hbdH*_GA_LHA_Rv | AGGAGACCCCATGATCGAGTTCGCCCTGCACGGCCACGTCGC |  |
| pLO3_KO_*hbdH*_GA_RHA_Fw | ACTCGATCATGGGGTCTCCTTCCGTGTCGTTCTTGAATGC | Primers used for amplification of the *hbdH* downstream region |
| pLO3_KO_*hbdH*_GA_RHA_Rv | TTTAAACAGTCGACTCTAGAGACCCGCTGTCCACCGTGGAGCTGG |  |
| pLO3_*hbdH*_KO_HA_seq_out_Fw | GCGTGTATTCGTCGATGAAGAAGC | Primers used for sequencing outside the *hbdH* coding region |
| pLO3_*hbdH*_KO_HA_seq_out_Rv | pLO3_mmsA3_KO_HA_seq_in_Rv |  |
| pLO3_*hbdH*_KO_HA_seq_in_Fw | pLO3_C3_KO_HA_seq_in_Fw | Primers used for sequencing inside the *hbdH* coding region |
| pLO3_*hbdH*_KO_HA_seq_in_Rv | CATACGCTGACCGTATTCGACCTG |  |
| pLO3_KO_*prpRBCMD*_GA_LHA_Fw | AGATCCTTTTAATTCGAGCTCGGATTTGCCGGCACCATCGGCCAC | Primers used for amplification of the *prpRBCMD* upstream region |
| pLO3_KO_*prpRBCMD*_GA_LHA_Rv | GGGCGATAACACCGCGCTGGCATTTACCCGGCTG |  |
| pLO3_KO_*prpRBCMD*_GA_RHA_Fw | CCAGCGCGGTGTTATCGCCCGGGCAACTTCATGC | Primers used for amplification of the *prpRBCMD* downstream region |
| pLO3_KO_*prpRBCMD*_GA_RHA_Rv | TTTAAACAGTCGACTCTAGATTGTCGATCTTGTCGATGTCCCAC |  |
| pLO3_*prpRBCMD*_KO_HA_seq_out_Fw | CACCGATCTATCACTCAATGTGACG | Primers used for sequencing inside the *prpRBCMD* coding region |
| pLO3_*prpRBCMD*_KO_HA_seq_out_Rv | GTTGCCGTAGGCGTAGAGCAC |  |
| pLO3_*prpRBCMD*_KO_HA_seq_in_Fw | CTCGGACATGTATTCGTCGATCAGC | Primers used for sequencing inside the *prpRBCMD* coding region |
| pLO3_*prpRBCMD*_KO_HA_seq_in_Rv | GGATCACGTCGACGTTCTTGAACG |  |
| pBBR1MCS-2-P*_phaC_ – mmsA1*_Fw | TGACATATGAGCATCGCAGAAAACCGGCAAC | Primers used for amplification of the *mmsA1* gene |
| pBBR1MCS-2-P*_phaC_ – mmsA1*_Rv | CTAACTAGTTTACTTCGCCACCGGCATGGTGAAC |  |
| pBBR1MCS-2-P*_phaC_ – mmsA2*_Fw | TGACATATGCAGACCATCATCGGCCATAGCATC | Primers used for amplification of the *mmsA2* gene |
| pBBR1MCS-2-P*_phaC_ – mmsA2*_Rv | CTAACTAGTTTATTTCGCCACCGGCATGGTGAAC |  |
| pBBR1MCS-2-P*_phaC_ – mmsA3*_Fw | TGACATATGAGTGCCGTGCCCAAGACC | Primers used for amplification of the *mmsA3* gene |
| pBBR1MCS-2-P*_phaC_ – mmsA3*_Rv | CTAACTAGTTTACTTCAGGCTGATGGTGGTGTTC |  |
| FW029_DNA_Cn1f | CGGCGTCATGAAGTACAAGG | Primers used to test for DNA contamination in total RNA preparations |
| FW030_DNA_Cn1r | CCTTCAGGCCCTCATACACC |  |
| FW031_DNA_Cn2f | CCTGCCGGCCTTCAACGTG | Primers used to test for DNA contamination in total RNA preparations |
| FW032_DNA_Cn2r | CTTCTTCGCCGGCTTCACC |  |
| pMTL71301-P*_acaD_*_Fw | CAGGAAACAGCTATGACCGCGGCCGCCACTGCCTTTTTCCATGGCGCTGG | Primers used for amplification of the *acaD* promoter |
| pMTL71301-P*_acaD_*_Rv | CGATATGCATATGGCAGATGAGGGTGGGGGTTTGGA |  |
| pMTL71301-P*_acaD_-hbdh*_Fw | CTCATCTGCCATATGCATATCGCCTTCATCGGCCTC | Primers used for amplification of the *hbdh* gene |
| pMTL71301-P*_acaD_-hbdh*_Rv | TCAGTTATCTAGATCCGGTGGATCCTCATTGCTTGCCCTCCTTGTCGGC |  |
